# Curcumin and Carnosic Acid Cooperate to Inhibit Proliferation and Alter Mitochondrial Function of Metastatic Prostate Cancer Cells

**DOI:** 10.3390/antiox10101591

**Published:** 2021-10-11

**Authors:** Saniya Ossikbayeva, Marina Khanin, Yoav Sharoni, Aviram Trachtenberg, Sultan Tuleukhanov, Richard Sensenig, Slava Rom, Michael Danilenko, Zulfiya Orynbayeva

**Affiliations:** 1 Department of Surgery, Drexel University College of Medicine, Philadelphia, PA 19102, USA; ossikbayeva.s@kaznmu.kz (S.O.); zulfiya.orynbayeva@crl.com (Z.O.); 2 Department of Biophysics and Biomedicine, Al-Farabi Kazakh National University, Almaty 050040, Kazakhstan; sultan.tuleuhanov@kaznu.kz; 3 Department of Clinical Biochemistry and Pharmacology, Faculty of Health Sciences, Ben-Gurion University of the Negev, Be’er Sheva 8410501, Israel; hanin@bgu.ac.il (M.K.); yoav@bgu.ac.il (Y.S.); aviramtr@post.bgu.ac.il (A.T.); 4 Department of Surgery, Cooper University Hospital, Camden, NJ 08103, USA; sensenigrb@verizon.net; 5 Department of Pathology and Laboratory Medicine, Lewis Katz School of Medicine, Temple University, Philadelphia, PA 19140, USA; srom@temple.edu

**Keywords:** prostate cancer, curcumin, carnosic acid, cell cycle, OxPhos, SGK1

## Abstract

Anticancer activities of plant polyphenols have been demonstrated in various models of neoplasia. However, evidence obtained in numerous in vitro studies indicates that proliferation arrest and/or killing of cancer cells require quite high micromolar concentrations of polyphenols that are difficult to reach in vivo and can also be (geno)toxic to at least some types of normal cells. The ability of certain polyphenols to synergize with one another at low concentrations can be used as a promising strategy to effectively treat human malignancies. We have recently reported that curcumin and carnosic acid applied at non-cytotoxic concentrations synergistically cooperate to induce massive apoptosis in acute myeloid leukemia cells, but not in normal hematopoietic and non-hematopoietic cells, via sustained cytosolic calcium overload. Here, we show that the two polyphenols can also synergistically suppress the growth of DU145 and PC-3 metastatic prostate cancer cell cultures. However, instead of cell killing, the combined treatment induced a marked inhibition of cell proliferation associated with G_0_/G_1_ cell cycle arrest. This was preceded by transient elevation of cytosolic calcium levels and prolonged dissipation of the mitochondrial membrane potential, without generating oxidative stress, and was associated with defective oxidative phosphorylation encompassing mitochondrial dysfunction. The above effects were concomitant with a significant downregulation of mRNA and protein expression of the oncogenic kinase SGK1, the mitochondria-hosted mTOR component. In addition, a moderate decrease in SGK1 phosphorylation at Ser422 was observed in polyphenol-treated cells. The mTOR inhibitor rapamycin produced a similar reduction in SGK1 mRNA and protein levels as well as phosphorylation. Collectively, our findings suggest that the combination of curcumin and carnosic acid at potentially bioavailable concentrations may effectively target different types of cancer cells by distinct modes of action. This and similar combinations merit further exploration as an anticancer modality.

## 1. Introduction

Historically, phytochemicals have been one of the major foundations of drug development [1]. Curcumin (CUR), the principal curcuminoid of the Indian spice turmeric (Supplementary Figure S1) has been the subject of numerous studies into its value as a cancer therapy that led to a number of clinical trials (e.g., [2,3,4]). The rationale for potential therapeutic applications of this compound is based on its pleiotropic effects on multiple cellular activities and regulatory pathways (see [4,5] for recent reviews), including the ability to induce oxidative stress [6,7,8,9,10] and/or endoplasmic reticulum stress [11,12,13,14] in various cancer cell types that lead to cell cycle arrest and cell death. However, the above effects are usually observed at high supraphysiological concentrations of CUR (≥ 10 µM) that are difficult to reach in vivo due to a low bioavailability and extensive metabolism of this polyphenol [15,16]. Furthermore, at such concentrations CUR has been found to induce geno/cytotoxicity to at least some types of normal cells [17,18,19,20,21].

Combinations of CUR with different phytochemicals or drugs have demonstrated enhanced anticancer effects in various models of human malignancies, as compared to single agents (see [22,23,24] for recent reviews). For instance, pairing CUR with the polyphenols quercetin [25], resveratrol [26,27], epigallocatechin gallate [28] or ursolic acid [29] resulted in synergistic inhibitory effects on the growth and survival of colorectal, breast and prostate cancer cells. Our previous study has demonstrated that the combination of CUR and the carotenoid lycopene synergistically inhibited androgen receptor signaling in prostate cancer cells [30]. Such a combinatory approach has the potential to overcome therapeutic limitations of plant polyphenols by minimizing their effective concentrations in synergistically acting combinations while maintaining or increasing anticancer efficiency.

We have recently shown that co-treatment of acute myeloid leukemia (AML) cells with CUR and carnosic acid (CA), a phenolic diterpene from rosemary (Supplementary Figure S1) [31], at non-cytotoxic concentrations of each compound (2.5–5.0 µM CUR + 5–10 µM CA) results in a rapid and massive cell death through the synergistic activation of both extrinsic and intrinsic apoptotic pathways [32,33]. Interestingly, in contrast to CA, other plant phenolic compounds, such as silibinin, rosmarinic acid, resveratrol, quercetin or parthenolide, did not cooperate with CUR in AML cells [32,34]. In the present study, we examined whether the two polyphenols (CUR and CA) applied at similar low concentrations (≤ 10 µM) would also synergize in inducing apoptotic cell death of DU145 and PC-3 cells, the most widely studied human metastatic prostate cancer cell lines [35]. Surprisingly, CUR + CA treatment caused only a slight or no induction of apoptosis in these cells; however, the combination dramatically inhibited clonal cell growth and G_1_-to-S cell cycle transition in a synergistic manner.

Development of primary tumors and their further dissemination to metastatic loci are shown to be accompanied by metabolic reprogramming towards advancement of oxidative processes and loss of apoptotic potential [36,37,38]. Recent discoveries in this field indicate an active involvement of mitochondria in cancer progression and the development of chemoresistance [39,40,41,42,43]. Various plant polyphenols have been shown to target mitochondria in cancer cells (reviewed in [44]). Therefore, here, we focused on characterizing the effects of CUR and CA, alone and in combination, on mitochondrial function in DU145 and PC-3 cells. The data demonstrated that the marked inhibition of cell proliferation by CUR + CA was preceded by dissipation of the mitochondrial membrane potential (Δψ_m_) and suppression of all respiratory enzyme complexes. Notably, these effects were not accompanied by intracellular accumulation of reactive oxygen species (ROS).

## 2. Materials and Methods

### 2.1. Materials

Curcumin (≥90%) and carnosic acid (≥95%) were purchased from Cayman Chemical (Ann Arbor, MI, USA) and Enzo Life Sciences, Inc. (Farmingdale, NY, USA), respectively. Stock solutions of both polyphenols were prepared in DMSO and were refreshed every two weeks. DMSO was used as a vehicle throughout all experiments at a final concentration of 0.2%.

### 2.2. Cell Lines

Metastatic human prostate cancer cells (DU145 and PC-3) were purchased from ATCC (Manassas, VA, USA) at the available passage 60 and used up to passage 70. Cells were maintained in RPMI 1640 medium supplemented with 10% FBS. The cells were grown at 37 °C in a humidified 5% CO_2_ atmosphere.

### 2.3. Alamar Blue Cell Viability Assay

Cells were seeded in a 96-well plate at a density of 9000 cells per well and treated with vehicle or polyphenols for 48 h. The cells were incubated with 100 μL of 3% Alamar Blue solution in a complete growth medium at 37 °C for 2 h [45]. The fluorescence signal of the Alamar Blue product resorufin (585 nm) was read on a BioTek Synergy 4 microplate reader (Winooski, VT, USA). In these and all the other experiments involving fluorescence, curcumin autofluorescence was subtracted from the signals obtained from cells treated with curcumin, alone and in combination with carnosic acid.

### 2.4. Assessment of Apoptosis

Apoptosis was evaluated using Annexin V-Propidium Iodide-based apoptosis kit (ThermoFisher Scientific, Waltham, MA, USA) and analyzed by flow cytometry on the BD Accuri C6 instrument. For each sample, 10,000 events were recorded. Annexin V-positive/PI-negative cells were considered to be in the early apoptotic phase; cells positive for both Annexin V and PI to be late apoptotic; and Annexin V-negative/PI-positive cells to be necrotic [46].

### 2.5. Colony Formation Assay

Clonogenic cell growth assay was performed with 20,000 PC-3 or DU145 cells seeded in a 6-well plate in the growth medium and incubated overnight. Cells were then treated with vehicle or polyphenols for 7 days. Colonies were fixed with 3.7% paraformaldehyde at room temperature for 5 min, rinsed with PBS, and stained with 0.05% crystal violet for 30 min. Cells were then washed with tap water and drained. The stained colonies were imaged on a Zeiss Axiovert 40 CFL inverted microscope with SPOT RT-SE™ digital camera (Diagnostic Instruments Inc., Sterling Heights, MI, USA) and analyzed using ImageJ program. Quantification of cell colonies per microscopic field of view was made using a density threshold.

### 2.6. Examination of Cell Cycle Distribution

Cells (1 × 10^6^) were synchronized in serum-free growth medium for 24 h (DU145) or 48 h (PC-3) and incubated with polyphenols for 24 h. After treatment, cells were washed with ice-cold PBS and fixed in 70% ethanol at −20 °C for 24 h. Cells were then rinsed twice with PBS and incubated in 1 mL of PBS containing 0.1% Triton X-100 and 50 μg of RNAse at room temperature for 30 min. Propidium iodide (10 μg/mL) was added to the cells for 20 min followed by fluorescence analysis in BD Accuri C6 flow cytometer (BD Biosciences, San Jose, CA, USA). For each sample, 10,000 events were recorded.

### 2.7. Preparation of Whole Cell Lysates and Western Blotting

Cells rinsed with PBS were lysed in ice-cold buffer containing 50 mM, HEPES, pH 7.5, 150 mM NaCl, 10% glycerol, 1% Triton X-100, 1.5 mM EGTA, 2 mM sodium orthovanadate, 20 mM sodium pyrophosphate, 50 mM NaF, 1 mM DTT and 1:50 cOmplete™ protease inhibitor cocktail (Sigma-Aldrich-Merck, Rehovot, Israel). The lysates were incubated for 10 min on ice and centrifuged at 20,000× *g*, 10 min, 4 °C. Supernatants (30 μg protein) were subjected to SDS-PAGE and blotted into nitrocellulose membrane (Whatman, Dassel, Germany). The membranes were blocked with 5% milk for 2 h and incubated with primary antibodies overnight at 4 °C, followed by incubation with HRP-conjugated secondary antibodies (Promega, Madison, WI, USA) for 1 h. The protein bands were visualized using Western Lightning™ Chemiluminescence Reagent Plus (PerkinElmer Life Sciences, Inc., Boston, MA, USA). The Integrated Density Value (IDV) of each protein band was quantitated using the Image Quant LAS 4000 system (GE Healthcare, Little Chalfont, UK). The following primary antibodies were used. Cyclin D1 (sc-6281), cyclin E (sc-481), CDK2 (sc-163), CDK4 (sc-260), p21^Cip1^ (sc-6246) and p27^Kip1^ (sc-1641) were purchased from Santa Cruz Biotechnology (Dallas, TX, USA). Phospho-SGK1 (Ser78; #5599) and SGK1 (#12103) were obtained from Cell Signaling Technology (Danvers, MA, USA), and phospho-SGK (Ser422; #SAB4503834) from Merck-Sigma-Aldrich (Rehovot, Israel). Calreticulin (sc-11398) or GAPDH (sc-47724) from Santa Cruz Biotechnology (Dallas, TX, USA) was used as the loading control.

### 2.8. Evaluation of Cytosolic Calcium Levels

Cells (0.2 × 10^6^) were trypsinized, washed with modified Krebs buffer (137 mM NaCl, 5 mM KCl, 1 mM KH_2_PO_4_, HEPES 20 mM, pH 7.4, 2 mM MgCl_2_, 2 mM CaCl_2_) and loaded with 2 μM Fluo-4AM at room temperature. After incubation for 15 min, cells were rinsed and kept in the buffer prior to measurements. For the Ca^2+^-free experiments, the buffer was prepared without CaCl_2_ and contained 1 mM EGTA. The changes in cytosolic calcium levels were analyzed for 30 min using BD Accuri C6 flow cytometer. For each sample, 1,000 events were recorded per each one-minute time point. To assess the maximal calcium level, 10 μM ionophore ionomycin was added in the end of each measurement.

### 2.9. Evaluation of the Mitochondrial Membrane Potential

Harvested cells (0.2 × 10^6^) were rinsed with modified Krebs buffer and loaded with 75 nM MitoRed (PromoCell GmbH, Heidelberg, Germany), the mitochondria membrane potential-sensitive indicator. After 30 min of incubation at room temperature, cells were rinsed and kept in the same buffer prior to examination. The signal was examined on a BD Accuri C6 flow cytometer. For the positive control, cells were treated with 2 μM FCCP (carbonyl cyanide-4-(trifluoromethoxy)phenylhydrazone), the dose which resulted in a collapse of the membrane potential. For each sample, 10,000 events were recorded.

### 2.10. Measurement of Oxidative Phosphorylation

Cellular respiration was analyzed at 37 °C using OROBOROS Oxygraph-2K (Innsbruck, Austria) [47,48]. Harvested cells were rinsed and resuspended in a modified Krebs buffer to assess the intact cells. Once the basal level of respiration was achieved, the ATPase inhibitor oligomycin (1 μg/mL) was added to evaluate the proton leak across the mitochondria inner membrane [49]. After inhibition, the 20 nM step FCCP titration was performed to “substitute” for the inhibited proton pump and stimulate respiration to its maximal rate. To examine OxPhos activity of individual respiratory enzymes, the permeabilized cell protocol was used. The addition of 10 μM digitonin compromises the plasma membrane enabling membrane impermeable modulators to enter cells and reach the mitochondria [50]. For this experiment, the cells were resuspended in the buffer, mimicking an intracellular environment (120 mM KCl, 10 mM NaCl, 1 mM KH_2_PO_4_, 20 mM MOPS, pH 7.2, 2 mM MgCl_2_, 1 mM EGTA, 0.7 mM CaCl_2_). The respiratory complexes were stimulated with 10 mM glutamate/2 mM malate (for complex I), 10 mM succinate (for complex II), 1 mM ascorbate/0.3 mM TMPD (for complex IV).

### 2.11. Measurement of the Levels of Reactive Oxygen Species

Harvested cells (0.2 × 10^6^) were rinsed with modified Krebs buffer and loaded with 2 µM CM-H_2_DCFDA, the indicator for cytosolic peroxides, or 5 µM MitoSox, the probe for mitochondrial superoxide (ThermoFisher Scientific, Waltham, MA, USA). After incubation for 30 min at room temperature, cells were rinsed with modified Krebs buffer and kept in the same buffer prior to examination. Fluorescence changes were analyzed on a BD Accuri C6 flow cytometer. H_2_O_2_ (100 µM) was used as a positive control in CM-H_2_DCFDA-loaded samples, and 2.5 μM antimycin A in MitoSox-loaded samples.

### 2.12. Reverse Transcription and Quantitative PCR

Total cell RNA was extracted using RNeasy Mini kit (QIAGEN) followed by treatment with Ambion^®^ Turbo DNase (ThermoFisher Scientific, Waltham, MA, USA). RNA quality and concentration were determined using a Nanodrop spectrophotometer (Nanodrop Technologies, Wilmington, DE, USA), and was adjusted to 50 ng/µL. RNA was converted to cDNA using the High-Capacity cDNA Reverse Transcriptase kit (Applied Biosystem, ThermoFisher Scientific, Waltham, MA, USA) and 1 µg RNA template, using Eppendorf Mastercycler Epigradient S (Hamburg, Germany). Initial gene profiling was performed on cDNA from DU145 cells using Human mTOR Signaling RT2 Profiler PCR Array (SA Biosciences, Qiagen, Germantown, MD, USA). GAPDH served as an internal control for gene expression normalization. To quantify gene expression in all cell lines, primers and TaqMan probes for SKG1 and GAPDH were acquired from Applied Biosystem (ThermoFisher Scientific, Waltham, MA, USA). qPCR was performed on ABI QuantStudio S3 real-time PCR system (ThermoFisher Scientific, Waltham, MA, USA). Data were analyzed with ABI DataAssist software (ThermoFisher Scientific, Waltham, MA, USA) using the 2^−∆∆Ct^ algorithm (relative quantification). Results are expressed in relative gene expression levels (fold regulation) compared with the untreated control. The qPCR was run in triplicate and repeated at least twice.

### 2.13. Statistical Analysis

Statistical analyses were performed using Prism GraphPad 7.0 software (San Diego, CA, USA). The cooperation between curcumin and carnosic acid was assessed by the Combination Index (CI) analysis using CompuSyn 1.0 software (ComboSyn Inc., Paramus, NJ, USA). The CI values were calculated on the basis of the levels of cell growth inhibition (fraction affected) by each agent individually and combination at non-constant ratios. CI values of <1, 1, and >1 show synergism, additivity and antagonism, respectively. Statistically significant differences between the means of several groups were assessed by one-way ANOVA with the Tukey multiple comparison post hoc analysis. The significance of the differences between two groups was estimated by unpaired, two-tailed Student’s *t*-test. Differences were considered significant at *p* < 0.05.

## 3. Results

### 3.1. Concentration-Dependent Effects of Curcumin, Carnosic Acid and Their Combinations on Cell Growth and Viability

**Figure 1:**
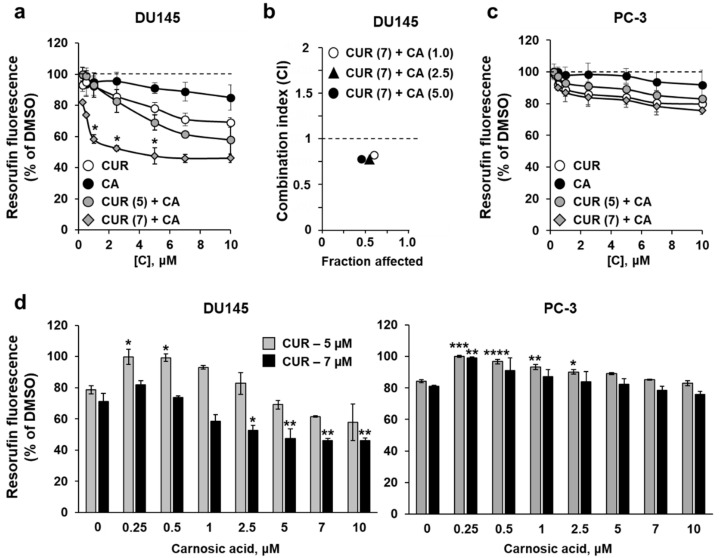
Effects of curcumin, carnosic acid and their combinations on the growth/viability of DU145 and PC-3 prostate cancer cells. (**a**,**c**) The Alamar Blue assay data obtained after 48 h of cell treatment with vehicle (DMSO) or the indicated concentrations of curcumin (CUR), carnosic acid (CA) and their combinations (CUR + CA). Values given in parentheses are concentrations (μM). Data are presented as mean ± SEM (*n* = 3). Statistically significant differences are indicated for CUR + CA vs. the sum of the effects of CUR and CA applied separately. * *p* < 0.05. (**b**) The Combination Index (CI) analysis of DU145 cell viability for the indicated CUR + CA (μM) combinations. The CI values are plotted against the levels of the fraction affected. (**d**) The Alamar Blue assay data demonstrating the effects of increasing concentrations of CA on DU145 and PC-3 cells treated with CUR at 5 µM or 7 µM. The values are derived from the data (mean ± SEM; *n* = 3) shown in panels (**a**,**c**). * *p* < 0.05; ** *p* < 0.01; *** *p* < 0.001 and **** *p* < 0.0001 vs. CUR alone (0 µM CA).

To evaluate the effects of curcumin (CUR) and carnosic acid (CA) on DU145 and PC-3 cells, we first employed the Alamar Blue assay to assess relative changes in the proportion of viable cells. Exposure to a range of (sub)micromolar concentrations of CUR (0.25–10 μM) for 48 h resulted in a dose-dependent decrease in cell growth/viability, with PC-3 cells being less responsive than DU145 cells (Figure 1a,c). When applied at the above concentrations, CA produced only minimal effects on both cell lines (Figure 1a,c). The data obtained in these experiments enabled us to select two relatively low concentrations of CUR (5 µM and 7 μM) for combined treatment with gradually increasing concentrations of CA. In DU145 cells, the combinations of 7 µM CUR and 1–5 µM CA produced a significantly enhanced reduction in the proportion of viable cells, as compared to the sum of the effects of single compounds (Figure 1a) or to the effect of CUR alone (Figure 1d). The combined effects of 5 µM CUR and CA were less pronounced, without an evidence for significant enhancement of the CUR effect by CA. Interestingly, when applied at lower concentrations (0.25–1.0 µM), CA even abolished the inhibitory effect of 5 µM CUR (Figure 1a,d). Detailed assessment of the cooperativity between the two polyphenols using the Combination Index (CI) analysis revealed a clear synergistic interaction between CUR and CA (CI < 1) at 7 μM CUR combined with 1–5 μM CA (Figure 1b), while the effects of 5 µM CUR combined with CA were additive at most (not shown). Therefore, the combination of 7 µM CUR and 5 µM CA, which produced the strongest synergistic reduction in cell viability (~50% compared to DMSO), was chosen for further studies. Surprisingly, these experiments showed no evidence of cooperation between the two polyphenols for PC-3 cells, i.e., the effects of CUR + CA combinations were similar to, or even weaker than, those of CUR alone. Again, the addition of CA at lower concentrations significantly abrogated the effects of CUR (Figure 1c,d).

**Figure 2:**
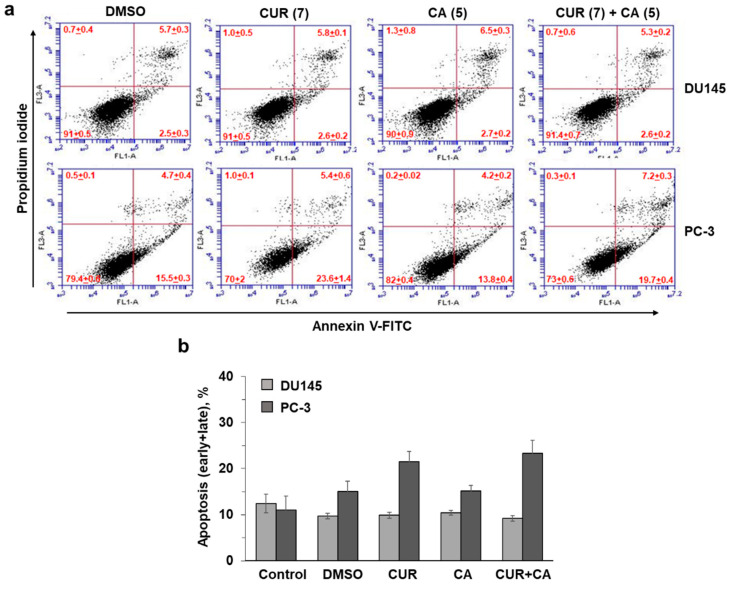
Effects of curcumin and carnosic acid on the induction of apoptosis. (**a**) Examples of primary flow cytometric data of annexin-V and propidium iodide binding to DU145 and PC-3 cells under the indicated treatment conditions (48 h). (**b**) Summarized data of apoptosis (early + late) induction, as exemplified in panel (**a**). Values given in parentheses are concentrations (μM). Data are presented as mean ± SEM (*n* = 3).

To examine whether the CUR ± CA-induced decreases in the relative quantity of viable cells were due to increased cell death, we evaluated the effects of the polyphenols using the annexin-V/PI assay in both DU145 and PC-3 cells. The results demonstrated that following 48 h of incubation, 7 µM CUR and 5 µM CA, alone or together, had a minimal or no apoptotic or necrotic effect on either cell line (Figure 2). For instance, treatment of PC-3 cells with CUR or CUR + CA resulted in a ≤10% increase in apoptotic cell death (Figure 2b), as compared to DMSO-treated cells (*p* = 0.208 or *p* = 0.069, respectively). This is unlike the previously observed rapid induction of massive apoptotic cell death in CUR + CA-treated AML cells [32,33].

**Figure 3:**
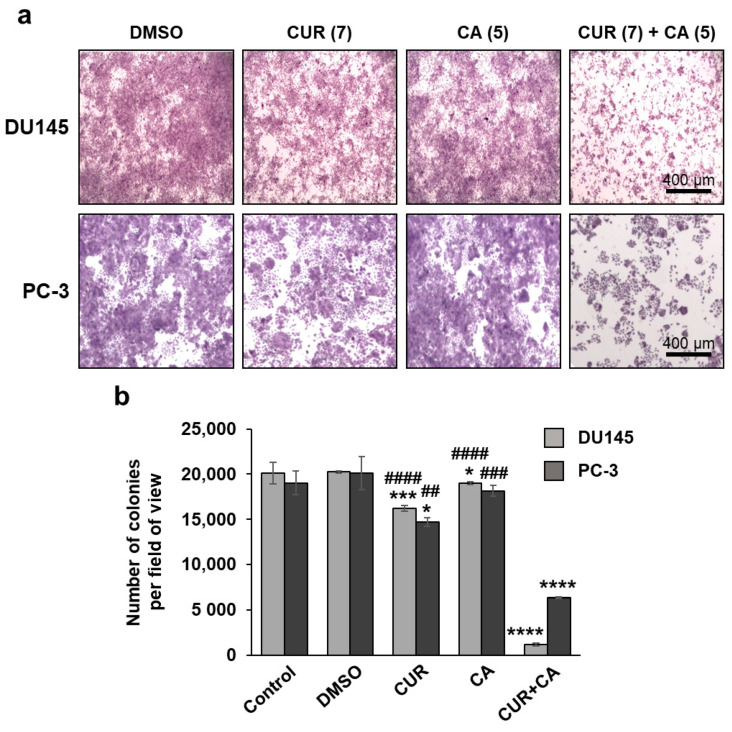
Colony formation analysis of the effects of curcumin and carnosic acid on cell growth. (**a**) Representative images of Crystal Violet-stained cell colonies obtained after 7 days of cell exposure to the indicated concentrations (in μM) of curcumin (CUR), carnosic acid (CA) and their combination (CUR + CA). Magnification: 40×; scale bar: 400 µm. (**b**) Quantitative evaluation of the colony formation data. The data are presented as averaged numbers of colonies per microscopic field of view (mean ± SEM; *n* = 3). Statistically significant differences for CUR, CA or CUR + CA vs. DMSO (* *p* < 0.05; *** *p* < 0.001 and **** *p* < 0.0001) and for CUR or CA applied separately vs. CUR + CA (## *p* < 0.01; ### *p* < 0.001 and #### *p* < 0.0001).

On the other hand, using the colony formation assay, we obtained strong support for the antiproliferative activity of the polyphenol combination in both cell lines tested. As shown in Figure 3, incubation of DU145 cells with CUR + CA for 7 days lead to an almost complete abrogation of clonal cell growth, whereas treatment with CUR or CA alone produced only a minor or no effect, respectively, as compared to untreated or vehicle-treated cells. Remarkably, while exhibiting no cooperativity in PC-3 cells when applied for 48 h (Figure 1c), the two polyphenols produced a marked synergistic suppression of clonal growth following 7 days of incubation (Figure 3), though the effect was somewhat less pronounced than that observed in DU145 cells.

### 3.2. Curcumin and Carnosic Acid Cooperate in Inducing Cell Cycle Arrest

**Figure 4:**
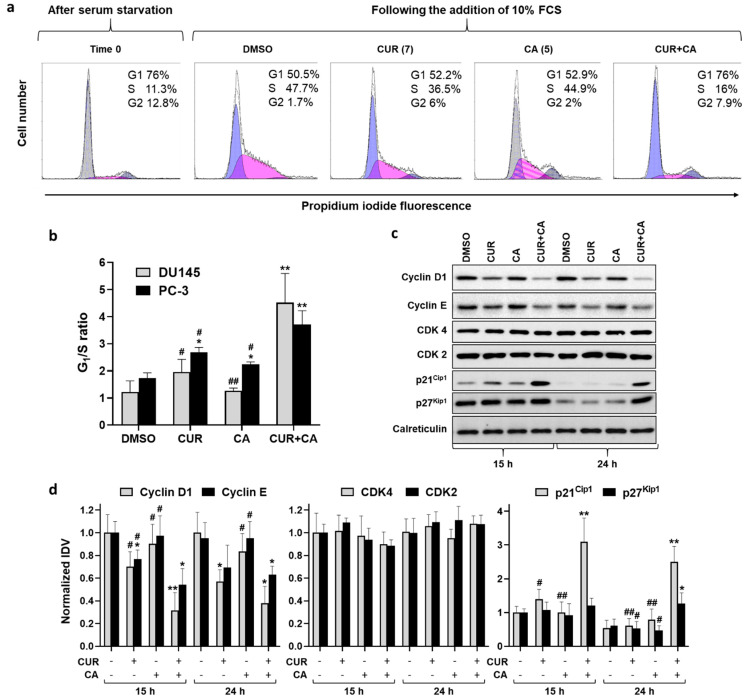
Modulation of cell cycle by curcumin, carnosic acid and their combination. (**a**) Representative flow cytometric data demonstrating inhibition of cell cycle progression in DU145 cells under the indicated treatment conditions (24 h). Values given in parentheses are concentrations (μM). (**b**) Quantitative data showing inhibition of G_1_/S cell cycle transition. (**c**) Expression profile of cell cycle regulatory proteins. (**d**) Quantitative analysis of the protein expression data. Integrated Density Values (IDVs) of the indicated protein bands normalized to IDVs of respective calreticulin bands are shown. All IDV ratios are relative to that of the control (DMSO) sample at 15 h assumed as 1.0. Data are presented as mean ± SD (*n* = 3). Statistically significant differences for CUR, CA or CUR + CA vs. DMSO (* *p* < 0.05 and ** *p* < 0.01) and for CUR or CA vs. CUR + CA (# *p* < 0.05 and ## *p* < 0.01) determined separately at 15 h and 24 h.

To further characterize the cooperative antiproliferative activity of CUR and CA, we evaluated the effects of the polyphenols, alone or together, on cell cycle distribution following cell synchronization at G_1_/S boundary by serum starvation. As exemplified in Figure 4a, incubation of synchronized DU145 cells with vehicle in 10% FBS-containing medium for 24 h resulted in a marked stimulation of G_1_-to-S cell cycle progression. Treatment with 7 µM CUR or 5 µM CA resulted in a moderate decrease in the proportion of S phase without a noticeable accumulation of cells in the G_0_/G_1_ phase. However, the combined treatment produced a dramatic G_0_/G_1_ cell cycle arrest. The averaged data presented in Figure 4b demonstrate a significantly greater increase in the G_1_/S ratio (indicative of G_0_/G_1_ arrest) by the combination compared to CUR or CA alone. Similar, but less pronounced effects were obtained in PC-3 cells (Figure 4b).

The G_0_/G_1_ cell cycle arrest induced by CUR + CA was accompanied by changes in the levels of several regulators of the G1-to-S transition, as determined in DU145 cells (Figure 4c and Supplementary Figure S2). Exposure to CUR and, especially, to its combination with CA for 15 h or 24 h resulted in an appreciable decrease in the level of D1 and E cyclins. The levels of CDK4 and CDK2 were not affected; however, those of the CDK inhibitors p21^Cip1^ and p27^Kip1^ were markedly elevated following combined treatments (Figure 4c,d).

### 3.3. Curcumin, Carnosic Acid and Their Combination Induce a Transient Rise of Cytosolic Calcium Levels

**Figure 5:**
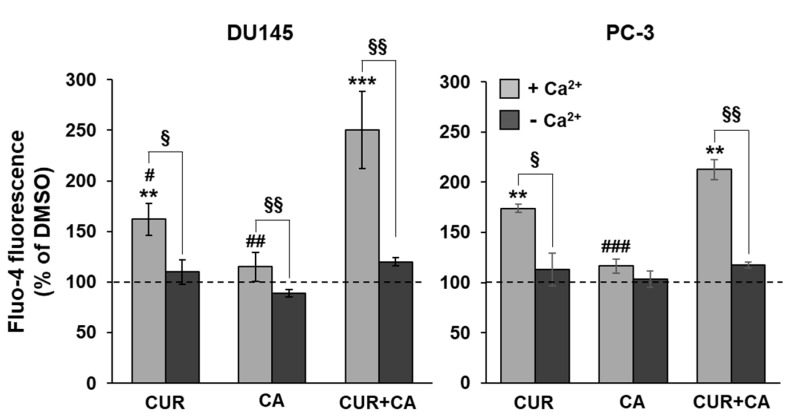
The combination of polyphenols induces elevation of cytosolic calcium levels in prostate cancer cells. Fluo-4 emission was recorded kinetically at 1 min intervals, starting immediately upon the addition of 7 μM CUR, 5 μM CA or their combination (CUR + CA). The bars show peak [Ca^2+^]_cyt_ signals recorded at 6–8 min (as in Supplementary Figure S3) relative to DMSO-treated cells. Data are presented as mean ± SEM (*n* = 3). Statistically significant differences for CUR, CA or CUR + CA vs. DMSO (** *p* < 0.01 and *** *p* < 0.001) and for CUR and CA vs. CUR + CA (# *p* < 0.05; ## *p* < 0.01 and ### *p* < 0.001). § *p* < 0.05 and §§ *p* < 0.01, significant difference between the two indicated groups (Student’s *t*-test).

We have recently reported that in AML cells, CUR + CA-induced apoptosis is associated with a sustained elevation of cytosolic calcium levels ([Ca^2+^]_cyt_) [33]. Here, we also found that in prostate cancer cells this combination also evoked a [Ca^2+^]_cyt_ rise to higher levels than those observed after single treatments (Figure 5). However, in contrast to leukemia cells, the [Ca^2+^]_cyt_ elevation was transient and moderate, reaching only 30–40% of the maximal signal provoked by 10 μM calcium ionophore ionomycin (Supplementary Figure S3). Further, while in CUR+CA-treated AML cells, calcium was primarily mobilized from the endoplasmic reticulum [33], in prostate cancer cells it mainly influxed from the extracellular space since the use of Ca^2+^ free buffer resulted in an 80–90% decrease in the magnitude of the calcium signal (Figure 5).

### 3.4. Effects of Polyphenols on Mitochondrial Functions

**Figure 6:**
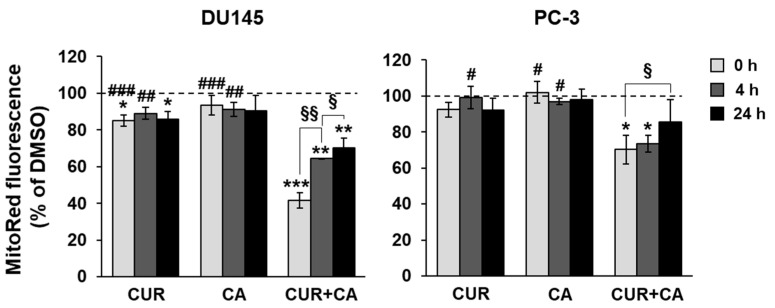
Combinatory effects of curcumin and carnosic acid on the mitochondrial membrane potential. Cells were treated with 7 μM CUR and 5 μM CA, alone and in combination (CUR + CA). The data were recorded at 5 min (0 h), 4 h and 24 h after the addition of DMSO or polyphenols. Data are presented as mean ± SEM (*n* = 3) relative to DMSO-treated cells. Statistically significant differences for CUR, CA or CUR + CA vs. DMSO (* *p* < 0.05; ** *p* < 0.01 and *** *p* < 0.001) and for CUR and CA vs. CUR + CA (# *p* < 0.05; ## *p* < 0.01 and ### *p* < 0.001). § *p* < 0.05 and §§ *p* < 0.01, significant difference between the indicated groups (Student’s *t*-test).

A modest elevation of calcium in the cytosol is sufficient to initiate calcium transport to the mitochondria [51]. The mitochondrial calcium uniport is an electrogenic process that occurs at the cost of the mitochondrial membrane potential (Δψ_m_). As shown in Figure 6, the addition of CUR or CA alone resulted in a slight reduction in Δψ_m_ in DU145 cells and was practically ineffective in PC-3 cells. However, the combination of these polyphenols markedly lowered the membrane potential to about 40% of the control level in DU145 cells, and to ~70% in PC-3 cells (Figure 6). This was a transient effect, echoing the transient cytosolic calcium elevation, as exemplified for DU145 cells in Supplementary Figure S3. The Δψ_m_ dropped within seconds after addition of the combination (0 h point in Figure 6) and partially recovered in 4–24 h.

**Figure 7:**
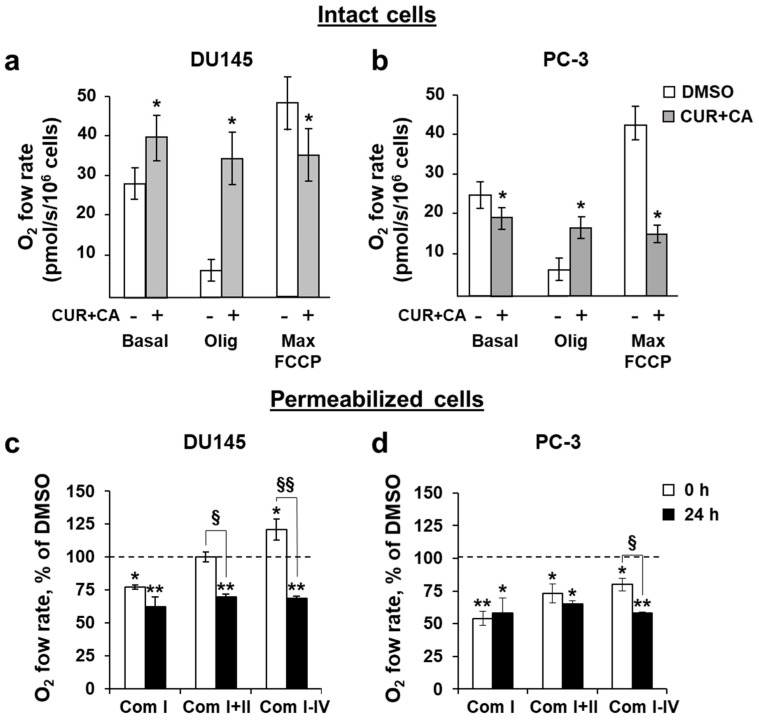
Combinatory effects of curcumin and carnosic acid on OxPhos. (**a**,**b**) Oxygen consumption in the presence or absence of 7 μM CUR and 5 μM CA (CUR + CA). Abbreviations: Basal, basal respiration; Olig, oligomycin-inhibited respiration; Max FCCP, the maximal respiratory capacity of mitochondria. (**c**,**d**) Respiration of permeabilized cells. The state 3 rates of OxPhos of complexes I, I+II, and I-IV measured upon addition (0 h) and after incubation (24 h) with the combination of CUR and CA, as compared to DMSO-treated cells. Data are presented as mean ± SEM (*n* = 3). Statistically significant differences in intact cell groups (**a**,**b**) treated with CUR + CA vs. DMSO: * *p* < 0.05. In permeabilized cell groups (**c**,**d**), statistically significant differences for the indicated groups vs. DMSO: * *p* < 0.05 and ** *p* < 0.01). § *p* < 0.05 and §§ *p* < 0.01, significant difference between the two indicated groups (Student’s *t*-test).

The alterations in Δψ_m_, which are determinant of the electron transport, prompted us to evaluate the effects of polyphenols on oxidative phosphorylation (OxPhos). We applied the protocol of sequential addition of oligomycin, an inhibitor of ATPase, followed by titration of the protonophore FCCP to explore possible mitochondrial damage in intact (non-permeabilized) cells [48]. The original respirograms are available in the Supplementary Materials (Figure S4). The addition of the CUR + CA combination stimulated mitochondrial respiration in DU145 cells (Figure 7a) but decreased it in PC-3 cells (Figure 7b). Oligomycin did not strongly inhibit the respiration in DU145 cells. Subsequent addition of FCCP was not able to further stimulate respiration as it would under normal conditions, meaning that in the presence of the combination of polyphenols, the mitochondria function at their maximal respiratory capacity. These data correlate with a decrease in the membrane potential (Figure 6), indicating uncoupling of OxPhos in DU145 cells. On the other hand, the combination of polyphenols decreased the respiration in PC-3 cells (Figure 7b), so almost no further inhibition was produced by oligomycin, and no stimulation was induced by FCCP. Overall, the above data demonstrate that the presence of CUR + CA prevents the modulatory action of oligomycin and FCCP on OxPhos regardless of the mode of the mitochondrial respiratory response to the polyphenols, i.e., enhancement in DU145 cells or suppression in PC-3 cells (Figure 7a,b). These results suggest that the possible mechanisms of CUR + CA effects on the mitochondrial respiration include a protonophoric activity of the combination.

The activities of individual respiratory complexes were evaluated using the cells permeabilized with the non-ionic detergent digitonin (Figure 7c,d) [50]. The integrated state 3 activities of the complexes I (Com I), I and II (Com I–II), and all complexes (Com I–IV) were assessed. Under permeabilized conditions, the contribution of calcium flow observed above is discounted, since in permeabilized cells the cytosolic content is diluted and, thus, the homeostatic integrity is compromised. This setting enables us to evaluate the potential direct effects of polyphenols on mitochondrial enzymes beyond plasma membrane-mediated calcium signaling.

In DU145 cells, the Com I activity was decreased by ~25% immediately (0 h) upon addition of the polyphenols, whereas the combined activities of Com I+II did not change significantly compared to DMSO-treated cells (Figure 7c). This is probably because of a higher rate of Com II respiration, as it is an electroneutral transporter and, therefore, it is less affected by membranotropic agents such as CUR. The combinatory activity of all complexes (Com I-IV) was elevated by ~20% over the control in agreement with a decrease in Δψ_m_ (Figure 6) and stimulated respiration observed in intact cells (Figure 7a). Following combined treatment of DU145 cells for 24 h, the OxPhos activities of all complexes were similarly reduced to the level of 60–70% of the control consistent with a decrease in Δψ_m_.

In PC-3 cells, the combination of polyphenols instantly (0 h) inhibited the activity of Com I to ~50%, of Com I+II to ~70% and of Com I-IV to ~80% of the control level (Figure 7d). These data are in consistence with the inhibited respiration observed in intact cells (Figure 7b**)**. Following 24 h of incubation, the functionality of all PC-3 mitochondrial complexes constituted only about 60% of the control, also echoing a decreased Δψ_m_ at 24 h. Thus, regardless of differences in the initial responses of the mitochondria to the CUR + CA combination, in 24 h the resulting outcome in both DU145 and PC-3 cells was a decreased capacity of OxPhos (Figure 7c,d). The inhibitory effect of CUR + CA on Com I activity was common for the two cell lines (Figure 7c,d). One explanation for this finding is that the polyphenols may potentially interact with this complex in prostate cancer cells.

### 3.5. Curcumin and Carnosic Acid Do Not Provoke Oxidative Stress in Prostate Cancer Cells

**Figure 8:**
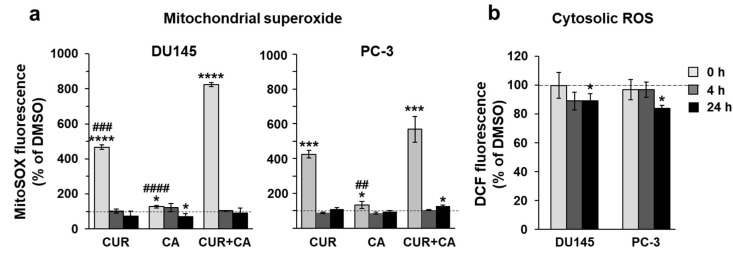
Effects of polyphenols on cellular production of reactive oxygen species. (**a**) Transient mitochondrial superoxide generation, as detected using the MitoSox probe following treatment with 7 μM CUR and 5 μM CA, alone and in combination (CUR + CA); (**b**) Cytosolic ROS assessed with the CM-H_2_DCFDA probe in CUR + CA-treated cells. Changes in fluorescence were recorded at 5 min (0 h), 4 h and 24 h after the addition of DMSO or polyphenols. Data are presented as mean ± SEM (*n* = 3) relative to DMSO-treated cells. Statistically significant differences (**a**) for CUR, CA or CUR + CA vs. DMSO (* *p* < 0.05; *** *p* < 0.001 and **** *p* < 0.0001) and for CUR and CA vs. CUR + CA (## *p* < 0.01, ### *p* < 0.001 and #### *p* < 0.0001). In panel (**b**), significant differences for the indicated groups vs. DMSO: * *p* < 0.05.

Retarded electron transport increases the chances of electron leakage and generation of superoxide [52]. We thus examined the levels of mitochondrial superoxide and cytosolic reactive oxygen species (ROS) in polyphenol-treated cells [53]. Within 5 min of the addition of CUR, alone or in combination with CA, a spike of superoxide signal was observed in both cancer cell lines (Figure 8a), which correlates with the drop in Δψ_m_ (Figure 6). This effect was transient and declined rapidly to the basal level, remaining so after 4 h and 24 h. CA alone had almost no effect on superoxide production but potentiated CUR-induced superoxide generation. In contrast to the mitochondrial superoxide, the cytosolic ROS levels did not significantly rise and even tended to slightly decrease with time (Figure 8b), suggesting that mitochondrial superoxide was effectively eliminated by endogenous scavengers preventing massive production of ROS.

### 3.6. Polyphenols Affect Mitochondria-Hosted mTOR Targets

**Figure 9:**
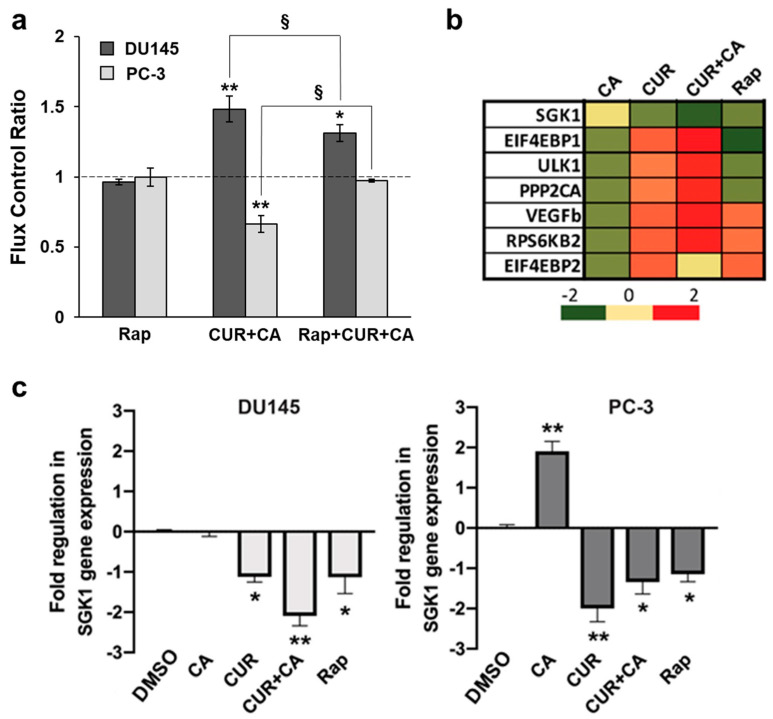
Effects of polyphenols and rapamycin on mitochondrial respiration and expression of mTOR downstream genes. (**a**) Rapamycin inhibits the effects of the polyphenol combination on mitochondrial respiration. Cells were pretreated with vehicle (DMSO) or 5 μM rapamycin (Rap) for 1 h followed by the addition of vehicle or 7 μM CUR and 5 μM CA (CUR + CA). Data are presented as mean ± SEM (*n* = 3) relative to DMSO. Statistically significant differences for the indicated groups vs. DMSO (* *p* < 0.05 and ** *p* < 0.01). § *p* < 0.05, significant differences between the indicated groups (Student’s *t*-test). (**b**) Changes in mTOR downstream transcriptional profile upon treatment of DU145 cells with 5 μM rapamycin or 7 μM CUR, 5 μM CA or CUR + CA) for 24 h, relative to DMSO. (**c**) Quantitative analysis of SGK1 gene expression. Data are presented as fold regulation vs. DMSO (mean ± SEM; *n* = 3), where DMSO is set as 0. Statistically significant differences for treatments vs. control (DMSO): * *p* < 0.05 and ** *p* < 0.01.

The mammalian target of rapamycin (mTOR) has been shown to regulate mitochondrial function, e.g., by interacting with or stimulating translation of mitochondrial proteins [54,55]. As both CUR [56,57] and CA [58,59] individually were found to affect mTOR and its downstream effectors in various cancer cell types, we hypothesized that the polyphenol-induced changes in the mitochondrial activities observed in our study (Figure 6 and Figure 7) may, at least in part, be related to the changes in mTOR signaling. To test this, we examined whether the effects of the CUR + CA combination on respiration of prostate cancer cells are influenced by rapamycin. Intact cells were incubated with 5 μM rapamycin [60] or vehicle for 1 h prior to addition of the combination of polyphenols. Due to lower rates of basal respiration in intact cells (Figure 7a,b), in order to demonstrate the cell responses to treatments the respiratory fluxes for each type of cells were normalized for their basal untreated respiration rates [61]. As shown in Figure 9a, rapamycin alone did not alter OxPhos in prostate cancer cells. However, the inhibitor caused a small but significant reduction in the stimulating effect of CUR + CA on DU145 respiration and prevented the drop in respiration in response to the combination in PC-3 cells (Figure 9a). The original respirograms are available in the Supplementary Materials (Figure S5).

The profile of the expression of mTOR genes that were affected by individual polyphenols, their combination, or rapamycin in DU145 cells after 24 h of treatment is shown in Figure 9b. The candidate genes that could physically be associated with mitochondria were searched among the transcripts of the mTOR downstream factors. Among the significantly altered genes, serum/glucocorticoid-regulated kinase 1 (SGK1) was the only one known to encode a protein localized in the mitochondrial outer membrane [62].

In DU145 cells, SGK1 gene expression was moderately down-regulated by rapamycin and CUR, and was not affected by CA, but when applied together the polyphenols caused a more pronounced downregulation of this tumor-promoting kinase (Figure 9b,c). In PC-3 cells, CA caused an elevation in SGK1 gene expression; however, CUR alone, the combination and rapamycin significantly reduced SGK1 expression (Figure 9c).

**Figure 10:**
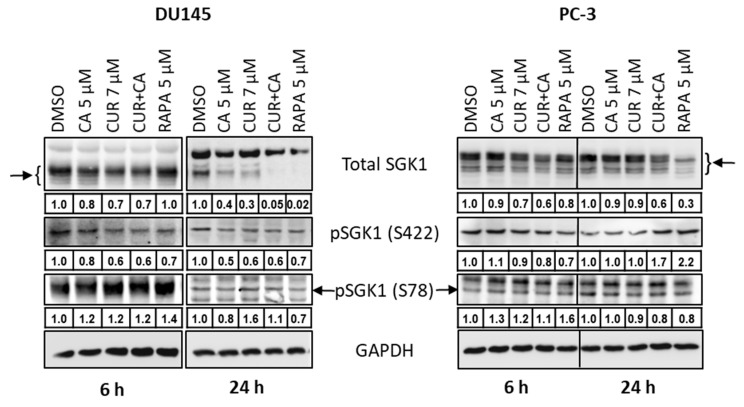
Effects of polyphenols and rapamycin on the protein expression and phosphorylation of serum- and glucocorticoid-regulated kinase 1 (SGK1) in prostate cancer cells. DU-145 and PC-3 cells were cultured with vehicle (DMSO), the indicated concentrations of curcumin (CUR) and carnosic acid (CA), alone and in combination, or the mTOR inhibitor rapamycin, for 6 or 24 h. Cells samples were then subjected to Western blot analysis. Integrated Density Values (IDVs) of the indicated protein bands normalized to IDVs of respective GAPDH bands are shown below corresponding blot images. Representative blots of 3 similar experiments are presented.

Consistent with the mRNA expression data (Figure 9b,c), treatment with the polyphenols, alone and in combination, or rapamycin resulted in a time-dependent reduction in SGK1 protein levels in both cell lines, as compared to DMSO-treated cells (Figure 10 and Supplementary Figure S6). Remarkably, in DU145 cells treated with CUR + CA or rapamycin for 24 h, SGK1 protein expression dropped to practically undetectable levels. In PC-3, the above treatments induced a moderate reduction in SGK1 levels (Figure 10).

The mTOR complex mTORC2 [63] and the 3-phosphoinositide-dependent protein kinase PDK2 [64] can activate SGK1 via phosphorylation at Ser422, and the mitogen-activated protein kinases (MAPKs) ERK5 [65] and p38 [66] via phosphorylation at Ser78. Here, we observed that treatment with CUR, CA or their combination resulted in a moderate reduction in SGK1 phosphorylation at Ser422 as compared to vehicle-treated cells. In DU145 cells, this effect was evidenced at both 6 h and 24 h, whereas in PC-3 cells a certain decrease in Ser422 phosphorylation was seen at 6 h, but not at 24 h (Figure 10 and Supplementary Figure S6). Interestingly, similar to the polyphenols, treatment with rapamycin also caused a decrease in Ser422 phosphorylation in both DU145 cells (at 6 h and 24 h) and PC-3 cells (at 6 h), implying that mTORC2, an indirect target of rapamycin [60,67,68], might be involved in the inhibitory effects of the polyphenols. The above treatments did not consistently affect SGK1 phosphorylation at Ser78, which mostly tended to increase slightly in the treated cells (Figure 10).

## 4. Discussion

The major finding of this study is that the plant polyphenols CUR and CA applied at low (<10 µM) concentrations, can synergistically cooperate to strongly suppress the growth of DU145 and PC-3 metastatic prostate cancer cell cultures in a time- and cell line-dependent manner. Notably, this effect was found to be essentially cytostatic (Figure 3), concomitant with G_0_/G_1_ cell cycle arrest (Figure 4), with only a negligible level of cell death (Figure 2). Inhibitory effects of various polyphenols, including CUR and CA, on cell cycle progression in cancer cells have been associated with the upregulation of both p21^Cip1^ and p27^Kip1^ (e.g., [69,70]). Importantly, some polyphenols, such as silibinin [71] or epigallocatechin gallate [72], were found to attenuate cellular degradation of these proteins, which was associated with cell cycle arrest, suggesting that a similar mechanism may, at least in part, account for the marked upregulation of p21^Cip1^ and p27^Kip1^ in CUR+CA-treated prostate cancer cells (Figure 4c,d).

The lack of cytotoxicity in CUR+CA-treated prostate cancer cells is strikingly different from the earlier-reported pronounced apoptotic cell death in AML cells [32,33,34]. Such distinct modes of CUR+CA action on prostate and blood cancer cells, coupled with the previously observed insusceptibility of untransformed hematopoietic and non-hematopoietic cells to this combination [32,33], indicate a remarkable cell-type dependence of the mechanisms underlying its anticancer effects. In AML cells, CUR+CA treatment results in a rapid (within 4–8 h) induction of apoptosis without inducing oxidative stress or changes in Δψ_m_ and is mediated solely by Ca^2+^ release from the endoplasmic reticulum leading to sustained [Ca^2+^]_cyt_ accumulation [33,34]. Although, similar to AML cells, CUR + CA treatment tended to lower cytosolic ROS in prostate cancer cells (Figure 8), prostate cancer cells exhibited a marked decrease in Δψ_m_ (Figure 6) and just a transient (within minutes) extracellular calcium-dependent [Ca^2+^]_cyt_ rise (Figure 5 and Figure S3). These cell type-dependent differences in regulatory responses might contribute to the observed distinct modes of CUR + CA action. Recently, Einbond et al. [73] have demonstrated that CUR (3.3–10.9) and CA (6.0–12.0 µM) can also cooperate in reducing the growth of triple-negative MDA-MB-468 human breast cancer cells in culture. However, this cooperation was evaluated only on the basis of the 3-(4,5-dimethyl-2-thiazol)-2,5-diphenyl-2H tetrazolilum bromide (MTT) assay, which is unable to distinguish changes in cell proliferation from changes in the extent of cell death. Therefore, the mode and the mechanism of action of CUR + CA on these cells are unclear.

In general, the mechanisms of synergistic cell growth-inhibitory effects of CUR + CA at low concentrations appear to differ from those underlying the effects of CUR (e.g., [8,9,11,14]) or CA [74,75,76,77] alone at higher concentrations (> 10 µM) in that the latter effects are usually found to be mediated by the induction of generalized cellular stress responses. Interestingly, Rodriguez-Garcia [9] reported that while the apoptotic effect of CUR on LNCaP and PC-3 prostate cancer cells was ROS-dependent and was associated with thioredoxin oxidation, the polyphenol silibinin, which reduced ROS levels and prevented thioredoxin oxidation in these cells, produced only a cytostatic effect. The latter finding supports, though indirectly, our data, showing that the cytostatic effect of CUR + CA on DU145 and PC-3 cells occurs in the absence of oxidative stress and is even associated with a slight reduction in the cytosolic ROS levels (Figure 7b).

Synergistic anticancer effects of various polyphenol combinations have been demonstrated in several tumor cell types (see [23,78] for recent reviews); however, the nature of the synergy between these compounds has not been fully elucidated. Several mechanisms of cooperation between antioxidant phytochemicals, including polyphenols, have been proposed (reviewed in [79]). For instance, individual components of a combination may target distinct signaling/transcriptional pathways or different proteins in the same cellular regulatory pathway. Furthermore, one of the components may help regenerate or chemically stabilize the other outside and/or inside the cell. The latter effects as well as the ability of certain phytochemicals, e.g., CA and CUR, to suppress drug efflux/multidrug resistance systems [80,81,82] may facilitate intracellular accumulation of one or both compounds. Indeed, Nimiya et al. [83] have shown that different antioxidants, including the plant phenolic compounds gallic, caffeic and rosmarinic acids, increased CUR stability in phosphate buffer and serum-free cell culture medium at physiological pH, as measured by colorimetric and HPLC assays. Consistent with these data, we have recently found that the addition of CA increases intracellular CUR levels in AML cells [34]. This was demonstrated by flow cytometry on the basis of CUR fluorescent properties [84]. In a recent study, Levine et al. [85] also showed that combined treatment of canine cancer cell lines with CA-rich rosemary extract and CUR-rich turmeric extract markedly increased intracellular CUR accumulation.

The data obtained in the present study indicate that unlike CUR, CA alone had primarily a minor or no significant influence on various cellular responses, such as changes in cell growth (Figure 1 and Figure 2), cell cycle distribution and regulatory protein levels (Figure 4), [Ca^2+^]_cyt_ levels (Figure 5 and Figure S3), mitochondrial superoxide production (Figure 8) and SGK1 gene expression (Figure 9). These results suggest that in metastatic prostate cancer cells, CA may act by potentiating CUR actions, likely by increasing its stability and/or cellular accumulation.

The metastatic ability of cancer cells is supported by the reprogramming of metabolic processes that include increases in the mitochondria membrane potential, rates of OxPhos, levels of ROS and calcium retention capacity [37,43]. Therefore, chemical agents that alter oxidative processes would perturb cancer metabolism and/or make neoplastic cells more susceptible to pharmacological factors. In this work, we specifically addressed the effects of CUR and CA on mitochondrial function. The immediate drop in Δψ_m_ observed in both prostate cancer cell lines treated with CUR + CA (Figure 6) could be associated with [Ca^2+^]_cyt_ elevation (Figure 5), which is pumped in the mitochondria at the cost of Δψ_m_. The mitochondria depolarization was likely the reason for prolonged suppression of all respiratory enzyme complexes, although initial respiratory responses of the two tested cancer cells to the combination of polyphenols differed—stimulation of the electron flow in DU145 cells and its inhibition in PC-3 (Figure 7). PC-3 cells were also less sensitive than DU145 cells to alterations of the calcium signal and Δψ_m_ caused by CUR + CA treatment (Figure 5 and Figure 6). The different responses of the mitochondria in the two prostate cancer cell lines to the polyphenols may be associated with their distinct metabolic features which include higher rates of glutamate/malate, citrate/malate and succinate oxidation and higher enzymatic activity of complex I in PC-3 cells compared to DU145 cells, as demonstrated in our previous study [43]. In addition, their mitochondrial membrane characteristics, e.g., variation of lipid content or the degree of saturation of acyl chains [86], may also differ. Earlier, using model membranes mimicking the mitochondria lipid bilayer we demonstrated a high affinity of CUR to cardiolipin, the mitochondria-unique phospholipid [87]. This in part explains the known curcumin’s protonophoric activity [88] and overall potential benefits in the treatment of broad range of metabolic diseases and conditions with key involvement of mitochondria. Still, several studies have demonstrated that in CUR-treated non-neoplastic and cancer cells, the polyphenol primarily localizes in the endoplasmic reticulum and lysosomes and only modestly accumulates in the mitochondria (e.g., [89,90]). These data suggest that the effects of CUR on the mitochondria are likely to be indirect. Of note, it has recently been suggested that physicochemical properties of polyphenols are responsible for their anticancer properties by virtue of their protonophoric and pro-oxidant properties rather than their specific effects on downstream molecular targets [44].

The dissimilar sensitivity of the two prostate cancer cell lines to the antiproliferative effects of CUR + CA could be related to the above differences in mitochondrial performances as well as to genetic features linked to their metastatic loci, such as brain (DU145) and bone (PC-3). Albeit sharing common malignant identities, the PC-3 cells were reported to have higher metastatic potential compared to DU145 cells [91]. Prolonged energetic stress caused by the combination of polyphenols in prostate cancer cells correlated with cell cycle arrest (Figure 4). However, dissipation of Δψ_m_ and altered oxidative phosphorylation did not lead to oxidative stress, since the massive increase in the mitochondrial superoxide signal right after addition of the combination of polyphenols, was quickly eliminated (Figure 8).

A key metabolic regulator, mTOR, plays a significant role in tumorigenesis and has been shown to be spatially associated with mitochondria and to control mitochondrial functionality [54,55,68]. As an instrumental tool, we employed rapamycin, a selective mTOR inhibitor which directly targets the mTORC1 complex and also indirectly blocks mTORC2 activity [60,67,68]. While rapamycin did not alter cellular respiration in our experimental setting, it moderately but significantly prevented the cell type-dependent effects of the CUR + CA combination, i.e., stimulation of respiration in DU145 cells and inhibition in PC-3 cells (Figure 9a), suggesting that these effects were partially mediated by mTOR.

Our search for a possible modulation of mitochondria-destined mTOR downstream targets by CUR and CA revealed SGK1, a multifunctional kinase which primarily localizes in the outer mitochondria membrane [62,92] and is implicated in regulating the growth, survival, cell cycle and apoptosis resistance of cancer cells [93]. Increased expression of SGK1 has been shown in myeloma [94], breast [95] and prostate [96] cancer cell cultures. Downregulation of SGK1 expression or inhibition of its kinase activity results in antiproliferative and cytotoxic effects on various types of malignant cells [93], including prostate cancer cells [96,97,98]. In prostate cancer, SGK1 inhibition also has anti-androgen effects [97].

To the best of our knowledge, only one publication related to the effect of CUR on SGK1 has been cited in MEDLINE/PubMed so far, which showed that treatment of renal carcinoma cells with 20 µM CUR did not affect either SGK1 protein levels or its phosphorylation [99]. No evidence of SGK1 modulation by CA has yet been reported. However, antiproliferative and cytotoxic effects of other plant phenolic compounds, such as resveratrol [100] and genistein [101], were found to correlate with SGK1 downregulation. Particularly, resveratrol inhibited SGK1 activity in hepatocellular carcinoma cells and also in a cell-free kinase assay. Moreover, silencing SGK1 enhanced resveratrol-induced inhibition of cell growth and apoptotic cell death, whereas SGK1 overexpression attenuated these effects [100]. By analogy, our finding that, similar to rapamycin, CUR ± CA suppressed mRNA and protein expression of SGK1 in DU145 and PC-3 cells (Figure 9b,c and Figure 10) suggests that SGK1 downregulation might contribute to the antiproliferative effect of these treatments, e.g., through upregulating p21^Cip1^ [94] and p27^Kip1^ [102]. There is accumulating evidence that SGK1 is an essential mediator of the phosphatidylinositol 3-kinase (PI3K)/mTOR signaling pathway (e.g., [93]). Thus, our finding that the polyphenols attenuate SGK1 phosphorylation at Ser422 (Figure 10), may suggest a role of the PI3K/mTOR pathway in the mechanism of the cytostatic effect of CUR + CA on prostate cancer cells. Further research is required to test this suggestion.

## 5. Conclusions

Our findings demonstrate that the combination of CUR and CA is more efficient than the individual compounds in arresting metastatic prostate cancer cell growth. The cytostatic effect of the combination was more pronounced in DU145 cells compared to PC-3 cells and was not accompanied by the induction of oxidative stress and cell death. CUR + CA-induced inhibition of cell growth was associated with G_0_/G_1_ cell cycle arrest and inhibition of mitochondrial function preceded by a rapid [Ca^2+^]_cyt_ rise and drop in Δψ_m_. Upon treatment with CUR±CA, the two cell lines mostly differed in the response magnitude and/or time course. Thus, while PC-3 cell growth was almost unaffected by CUR + CA at 48 h, the two cell lines responded similarly after 7 days of treatment (Figure 1 vs. Figure 3). Likewise, when compared to DU145 cells, PC-3 cells exhibited generally similar, though less pronounced changes in the cell cycle (Figure 4a), Ca^2+^_cyt_ (Figure 5), Δψ_m_ (Figure 6), superoxide and ROS levels (Figure 8) and SGK1 expression (Figure 9c and Figure 10). The main difference between DU145 and PC-3 cells was the dissimilar modulation of the mitochondrial respiration in response to polyphenol treatment (Figure 7 and Figure 9a), which may or may not relate to the different sensitivity of the two cell lines to the polyphenols.

Prostate cancer is the second most common cancer in men worldwide, mainly in countries with high Human Development Index [103,104]. Although most patients with localized disease have high survival rates, patients with metastatic prostate cancer have poor prognosis, with a 5-year survival rate of about 30%. Therefore, our findings presented here warrant further testing of this combination in translational studies that may lead to clinical development. Synergistically acting combinations of low concentrations of plant polyphenols or related agents with enhanced anti-cancer capacities may represent a safe and efficient way of dietary and/or pharmacological intervention in human malignancies, including prostate cancer. We believe that under prolonged energetic stress caused by the combination of CUR and CA, the cancer cells may become more vulnerable projecting a better response to chemotherapeutic and/or radiation treatments. Still, deeper research is required to elucidate the molecular mechanism of the synergistic effects of CUR and CA on cellular signaling and integrated metabolic pathways in order to establish polyphenol-based combinatory cancer therapeutics or adjuvants to conventional treatment modalities. Characterization of the mechanistic interactions between the mitochondrial energetic machinery and the mitochondria-resident SGK1 expression and activity would be of great interest per se in understanding how the confined transcriptional control is exerted locally over the mitochondrial functions.

## Data Availability

Data is contained within the article or supplementary material.

## Supplementary Materials

The following are available online at https://www.mdpi.com/article/10.3390/antiox10101591/s1. Figure S1: Chemical structures of curcumin and carnosic acid, Figure S2: Original images of the Western blots images shown in Figure 4c, Figure S3: Curcumin and carnosic acid cooperate in inducing moderate and transient cytosolic calcium elevation in DU145 prostate cancer cells, Figure S4: Combinatory effects of curcumin and carnosic acid on mitochondria respiration, Figure S5: Oxygraphic records of the effects of the combination of curcumin and carnosic acid on respiration of prostate cancer cells, Figure S6: Original images of the Western blots shown in Figure 10.

## Abbreviations

CA	Carnosic acid
CDK	Cyclin-dependent kinase
CM-H2DCFDA	5-(and-6)-chloromethyl-2′,7′-dichlorodihydrofluorescein diacetate, acetyl ester
CUR	Curcumin
DTT	Dithiothreitol
EGFR	Epidermal growth factor receptor
FBS	Fetal bovine serum
FCCP	Carbonyl cyanide-4-(trifluoromethoxy)phenylhydrazone
GAPDH	Glyceraldehyde phosphate dehydrogenase
HRP	Horseradish peroxidase
mTOR	Mammalian target of rapamycin
NFκB	Nuclear factor kappa B
OxPhos	Oxidative phosphorylation
ROS	Reactive oxygen species
SGK1	Serum/glucocorticoid regulated kinase 1
TMPD	N,N,N′,N′-Tetramethyl-p-phenylenediamine dihydrochloride

## References

[1] Newman D.J., Cragg G.M. (2020). Natural products as sources of new drugs over the nearly four decades from 01/1981 to 09/2019. J. Nat. Prod..

[2] Dhillon N., Aggarwal B.B., Newman R.A., Wolff R.A., Kunnumakkara A.B., Abbruzzese J.L., Ng C.S., Badmaev V., Kurzrock R. (2008). Phase II Trial of Curcumin in Patients with Advanced Pancreatic Cancer. Clin. Cancer Res..

[3] Choi Y.H., Han D.H., Kim S.-W., Kim M.-J., Sung H.H., Jeon H.G., Jeong B.C., Seo S.I., Jeon S.S., Lee H.M. (2019). A randomized, double-blind, placebo-controlled trial to evaluate the role of curcumin in prostate cancer patients with intermittent androgen deprivation. Prostate.

[4] Giordano A., Tommonaro G. (2019). Curcumin and Cancer. Nutrients.

[5] Mortezaee K., Salehi E., Mahyari H.M., Motevaseli E., Najafi M., Farhood B., Rosengren R.J., Sahebkar A. (2019). Mechanisms of apoptosis modulation by curcumin: Implications for cancer therapy. J. Cell. Physiol..

[6] Khan M.A., Gahlot S., Majumdar S. (2012). Oxidative Stress Induced by Curcumin Promotes the Death of Cutaneous T-cell Lymphoma (HuT-78) by Disrupting the Function of Several Molecular Targets. Mol. Cancer Ther..

[7] Woo J.-H., Kim Y.-H., Choi Y.-J., Kim D.-G., Lee K.-S., Bae J.H., Min D.S., Chang J.-S., Jeong Y.-J., Lee Y.H. (2003). Molecular mechanisms of curcumin-induced cytotoxicity: Induction of apoptosis through generation of reactive oxygen species, down-regulation of Bcl-XL and IAP, the release of cytochrome c and inhibition of Akt. Carcinogenesis.

[8] Larasati Y., Yoneda-Kato N., Nakamae I., Yokoyama T., Meiyanto E., Kato J.-Y. (2018). Curcumin targets multiple enzymes involved in the ROS metabolic pathway to suppress tumor cell growth. Sci. Rep..

[9] Garcia A.R., Hevia D., Mayo J.C., Gonzalez-Menendez P., Coppo L., Lu J., Holmgren A., Sainz R.M. (2017). Thioredoxin 1 modulates apoptosis induced by bioactive compounds in prostate cancer cells. Redox Biol..

[10] Wang L., Chen X., Du Z., Li G., Chen M., Chen X., Liang G., Chen T. (2017). Curcumin suppresses gastric tumor cell growth via ROS-mediated DNA polymerase γ depletion disrupting cellular bioenergetics. J. Exp. Clin. Cancer Res..

[11] Lee W.-J., Chien M.-H., Chow J.-M., Chang J.-L., Wen Y.-C., Lin Y.-W., Cheng C.-W., Lai G.-M., Hsiao M., Lee L.-M. (2015). Nonautophagic cytoplasmic vacuolation death induction in human PC-3M prostate cancer by curcumin through reactive oxygen species -mediated endoplasmic reticulum stress. Sci. Rep..

[12] Wang L., Wang L., Song R., Shen Y., Sun Y., Gu Y., Shu Y., Xu Q. (2011). Targeting Sarcoplasmic/Endoplasmic Reticulum Ca2+-ATPase 2 by Curcumin Induces ER Stress-Associated Apoptosis for Treating Human Liposarcoma. Mol. Cancer Ther..

[13] Kim B., Kim H.S., Jung E.-J., Lee J.Y., Tsang B.K., Lim J.M., Song Y.S. (2016). Curcumin induces ER stress-mediated apoptosis through selective generation of reactive oxygen species in cervical cancer cells. Mol. Carcinog..

[14] Rivera M., Ramos Y., Rodríguez-Valentín M., López-Acevedo S., Cubano L.A., Zou J., Zhang Q., Wang G., Boukli N.M. (2017). Targeting multiple pro-apoptotic signaling pathways with curcumin in prostate cancer cells. PLoS ONE.

[15] Dei Cas M., Ghidoni R. (2019). Dietary Curcumin: Correlation between Bioavailability and Health Potential. Nutrients.

[16] Sanchez M.A.N., González-Sarrías A., Vaquero M.R., Villalba R.G., Selma M.V., Tomas-Barberan F., García-Conesa M.-T., Espín J.C. (2015). Dietary phenolics against colorectal cancer-From promising preclinical results to poor translation into clinical trials: Pitfalls and future needs. Mol. Nutr. Food Res..

[17] Gautam S.C., Xu Y.X., Pindolia K., Janakiraman N., Chapman R.A. (1998). Nonselective Inhibition of Proliferation of Transformed and Nontransformed Cells by the Anticancer Agent Curcumin (Diferuloylmethane). Biochem. Pharmacol..

[18] Azqueta A., Collins A. (2016). Polyphenols and DNA Damage: A Mixed Blessing. Nutrients.

[19] Zikaki K., Aggeli I.-K., Gaitanaki C., Beis I. (2014). Curcumin induces the apoptotic intrinsic pathway via upregulation of reactive oxygen species and JNKs in H9c2 cardiac myoblasts. Apoptosis.

[20] Hollborn M., Chen R., Wiedemann P., Reichenbach A., Bringmann A., Kohen L. (2013). Cytotoxic Effects of Curcumin in Human Retinal Pigment Epithelial Cells. PLoS ONE.

[21] Fox J.T., Sakamuru S., Huang R., Teneva N., Simmons S., Xia M., Tice R.R., Austin C.P., Myung K. (2012). High-throughput genotoxicity assay identifies antioxidants as inducers of DNA damage response and cell death. Proc. Natl. Acad. Sci. USA.

[22] Vue B., Zhang S., Chen Q.-H. (2015). Synergistic Effects of Dietary Natural Products as Anti-Prostate Cancer Agents. Nat. Prod. Commun..

[23] Hosseini-Zare M.S., Sarhadi M., Zarei M., Thilagavathi R., Selvam C. (2021). Synergistic effects of curcumin and its analogs with other bioactive compounds: A comprehensive review. Eur. J. Med. Chem..

[24] Lin S.R., Chang C.H., Hsu C.F., Tsai M.J., Cheng H., Leong M.K., Sung P.J., Chen J.C., Weng C.F. (2020). Natural compounds as potential adjuvants to cancer therapy: Preclinical evidence. Br. J. Pharmacol..

[25] Kundur S., Prayag A., Selvakumar P., Nguyen H., McKee L., Cruz C., Srinivasan A., Shoyele S., Lakshmikuttyamma A. (2019). Synergistic anticancer action of quercetin and curcumin against triple-negative breast cancer cell lines. J. Cell. Physiol..

[26] Majumdar A.P.N., Banerjee S., Nautiyal J., Patel B.B., Patel V., Du J., Yu Y., Elliott A.A., Levi E., Sarkar F.H. (2009). Curcumin Synergizes With Resveratrol to Inhibit Colon Cancer. Nutr. Cancer.

[27] Gavrilas L.I., Cruceriu D., Ionescu C., Miere D., Balacescu O. (2019). Pro-apoptotic genes as new targets for single and combinatorial treatments with resveratrol and curcumin in colorectal cancer. Food Funct..

[28] Eom D.-W., Lee J.H., Kim Y.-J., Hwang G.S., Kim S.-N., Kwak J.H., Cheon G.J., Kim K.H., Jang H.-J., Ham J. (2015). Synergistic effect of curcumin on epigallocatechin gallate-induced anticancer action in PC3 prostate cancer cells. BMB Rep..

[29] Lodi A., Saha A., Lu X., Wang B., Sentandreu E., Collins M., Kolonin M.G., DiGiovanni J., Tiziani S. (2017). Combinatorial treatment with natural compounds in prostate cancer inhibits prostate tumor growth and leads to key modulations of cancer cell metabolism. NPJ Precis. Oncol..

[30] Linnewiel-Hermoni K., Khanin M., Danilenko M., Zango G., Amosi Y., Levy J., Sharoni Y. (2015). The anti-cancer effects of carotenoids and other phytonutrients resides in their combined activity. Arch. Biochem. Biophys..

[31] Bahri S., Jameleddine S., Shlyonsky V. (2016). Relevance of carnosic acid to the treatment of several health disorders: Molecular targets and mechanisms. Biomed. Pharmacother..

[32] Pesakhov S., Khanin M., Studzinski G.P., Danilenko M. (2010). Distinct Combinatorial Effects of the Plant Polyphenols Curcumin, Carnosic Acid, and Silibinin on Proliferation and Apoptosis in Acute Myeloid Leukemia Cells. Nutr. Cancer.

[33] Pesakhov S., Nachliely M., Barvish Z., Aqaqe N., Schwartzman B., Voronov E., Sharoni Y., Studzinski G.P., Fishman D., Danilenko M. (2016). Cancer-selective cytotoxic Ca2+ overload in acute myeloid leukemia cells and attenuation of disease progression in mice by synergistically acting polyphenols curcumin and carnosic acid. Oncotarget.

[34] Trachtenberg A., Muduli S., Sidoryk K., Cybulski M., Danilenko M. (2019). Synergistic Cytotoxicity of Methyl 4-Hydroxycinnamate and Carnosic Acid to Acute Myeloid Leukemia Cells via Calcium-Dependent Apoptosis Induction. Front. Pharmacol..

[35] Namekawa T., Ikeda K., Horie-Inoue K., Inoue S. (2019). Application of Prostate Cancer Models for Preclinical Study: Advantages and Limitations of Cell Lines, Patient-Derived Xenografts, and Three-Dimensional Culture of Patient-Derived Cells. Cells.

[36] Palmberg C., Rantala I., Tammela T.L., Helin H., Koivisto P.A. (2000). Low apoptotic activity in primary prostate carcinomas without response to hormonal therapy. Oncol. Rep..

[37] Freitas M., Baldeiras I., Proença T., Alves V., Mota-Pinto A., Sarmento-Ribeiro A. (2012). Oxidative stress adaptation in aggressive prostate cancer may be counteracted by the reduction of glutathione reductase. FEBS Open Bio.

[38] Koivisto P., Visakorpi T., Rantala I., Isola J. (1997). Increased cell proliferation activity and decreased cell death are associated with the emergence of hormone-refractory recurrent prostate cancer. J. Pathol..

[39] Kaambre T., Chekulayev V., Shevchuk I., Karu-Varikmaa M., Timohhina N., Tepp K., Bogovskaja J., Kütner R., Valvere V., Saks V. (2012). Metabolic control analysis of cellular respiration in situ in intraoperational samples of human breast cancer. J. Bioenerg. Biomembr..

[40] Koit A., Shevchuk I., Ounpuu L., Klepinin A., Chekulayev V., Timohhina N., Tepp K., Puurand M., Truu L., Heck K. (2017). Mitochondrial Respiration in Human Colorectal and Breast Cancer Clinical Material Is Regulated Differently. Oxidative Med. Cell. Longev..

[41] Martinez-Outschoorn U.E., Pestell R.G., Howell A., Tykocinski M.L., Nagajyothi F., Machado F.S., Tanowitz H.B., Sotgia F., Lisanti M.P. (2011). Energy transfer in “parasitic” cancer metabolism. Cell Cycle.

[42] Wallace D.C. (2012). Mitochondria and cancer. Nat. Rev. Cancer.

[43] Panov A., Orynbayeva Z. (2013). Bioenergetic and Antiapoptotic Properties of Mitochondria from Cultured Human Prostate Cancer Cell Lines PC-3, DU145 and LNCaP. PLoS ONE.

[44] Stevens J.F., Revel J.S., Maier C.S. (2018). Mitochondria-Centric Review of Polyphenol Bioactivity in Cancer Models. Antioxid. Redox Signal..

[45] Rampersad S.N. (2012). Multiple Applications of Alamar Blue as an Indicator of Metabolic Function and Cellular Health in Cell Viability Bioassays. Sensors.

[46] Galluzzi L., Vitale I., Abrams J.M., Alnemri E.S., Baehrecke E.H., Blagosklonny M.V., Dawson T.M., Dawson V.L., El-Deiry W.S., Fulda S. (2012). Molecular definitions of cell death subroutines: Recommendations of the Nomenclature Committee on Cell Death 2012. Cell Death Differ..

[47] Gnaiger E., Steinlechner-Maran R., Méndez G., Eberl T., Margreiter R. (1995). Control of mitochondrial and cellular respiration by oxygen. J. Bioenerg. Biomembr..

[48] Pesta D., Gnaiger E. (2012). High-resolution respirometry: OXPHOS protocols for human cells and permeabilized fibers from small biopsies of human muscle. Methods Mol. Biol..

[49] Brand M.D., Nicholls D.G. (2011). Assessing mitochondrial dysfunction in cells. Biochem. J..

[50] Kuznetsov A.V., Veksler V., Gellerich F.N., Saks V., Margreiter R., Kunz W.S. (2008). Analysis of mitochondrial function in situ in permeabilized muscle fibers, tissues and cells. Nat. Protoc..

[51] Duchen M.R. (2000). Mitochondria and calcium: From cell signalling to cell death. J. Physiol..

[52] Murphy M.P. (2009). How mitochondria produce reactive oxygen species. Biochem. J..

[53] Kalyanaraman B., Darley-Usmar V., Davies K.J., Dennery P.A., Forman H.J., Grisham M.B., Mann G.E., Moore K., Roberts L.J., Ischiropoulos H. (2012). Measuring reactive oxygen and nitrogen species with fluorescent probes: Challenges and limitations. Free Radic. Biol. Med..

[54] Ramanathan A., Schreiber S.L. (2009). Direct control of mitochondrial function by mTOR. Proc. Natl. Acad. Sci. USA.

[55] Morita M., Gravel S.-P., Hulea L., Larsson O., Pollak M., St-Pierre J., Topisirovic I. (2015). mTOR coordinates protein synthesis, mitochondrial activity and proliferation. Cell Cycle.

[56] Beevers C.S., Li F., Liu L., Huang S. (2006). Curcumin inhibits the mammalian target of rapamycin-mediated signaling pathways in cancer cells. Int. J. Cancer.

[57] Yu S., Shen G., Khor T.O., Kim J.H., Kong A.-N.T. (2008). Curcumin inhibits Akt/mammalian target of rapamycin signaling through protein phosphatase-dependent mechanism. Mol. Cancer Ther..

[58] El-Huneidi W., Bajbouj K., Muhammad J., Vinod A., Shafarin J., Khoder G., Saleh M., Taneera J., Abu-Gharbieh E. (2021). Carnosic Acid Induces Apoptosis and Inhibits Akt/mTOR Signaling in Human Gastric Cancer Cell Lines. Pharmaceuticals.

[59] Gao Q., Liu H., Yao Y., Geng L., Zhang X., Jiang L., Shi B., Yang F. (2015). Carnosic acid induces autophagic cell death through inhibition of the Akt/mTOR pathway in human hepatoma cells. J. Appl. Toxicol..

[60] Schieke S.M., Phillips D., McCoy J.P., Aponte A.M., Shen R.-F., Balaban R.S., Finkel T. (2006). The Mammalian Target of Rapamycin (mTOR) Pathway Regulates Mitochondrial Oxygen Consumption and Oxidative Capacity. J. Biol. Chem..

[61] Gnaiger E., Dykens J., Will Y. (2008). Polarographic oxygen sensors, the oxygraph, and high-resolution respirometry to assess mitochondrial functions. Drug-Induced Mitochondrial Dysfunction.

[62] Engelsberg A., Kobelt F., Kuhl D. (2006). The N-terminus of the serum- and glucocorticoid-inducible kinase Sgk1 specifies mitochondrial localization and rapid turnover. Biochem. J..

[63] García-Martínez J.M., Alessi D. (2008). mTOR complex 2 (mTORC2) controls hydrophobic motif phosphorylation and activation of serum- and glucocorticoid-induced protein kinase 1 (SGK1). Biochem. J..

[64] Kobayashi T., Cohen P. (1999). Activation of serum- and glucocorticoid-regulated protein kinase by agonists that activate phosphatidylinositide 3-kinase is mediated by 3-phosphoinositide-dependent protein kinase-1 (PDK1) and PDK2. Biochem. J..

[65] Hayashi M., Tapping R.I., Chao T.-H., Lo J.-F., King C., Yang Y., Lee J.-D. (2001). BMK1 Mediates Growth Factor-induced Cell Proliferation through Direct Cellular Activation of Serum and Glucocorticoid-inducible Kinase. J. Biol. Chem..

[66] Meng F., Yamagiwa Y., Taffetani S., Han J., Patel T. (2005). IL-6 activates serum and glucocorticoid kinase via p38α mitogen-activated protein kinase pathway. Am. J. Physiol. Cell Physiol..

[67] Sarbassov D.D., Ali S.M., Sengupta S., Sheen J.-H., Hsu P.P., Bagley A.F., Markhard A.L., Sabatini D.M. (2006). Prolonged Rapamycin Treatment Inhibits mTORC2 Assembly and Akt/PKB. Mol. Cell.

[68] Szwed A., Kim E., Jacinto E. (2021). Regulation and metabolic functions of mTORC1 and mTORC2. Physiol. Rev..

[69] Steiner M., Priel I., Giat J., Levy J., Sharoni Y., Danilenko M. (2001). Carnosic Acid Inhibits Proliferation and Augments Differentiation of Human Leukemic Cells Induced by 1,25-Dihydroxyvitamin D3 and Retinoic Acid. Nutr. Cancer.

[70] Srivastava R.K., Chen Q., Siddiqui I., Sarva K., Shankar S. (2007). Linkage of Curcumin-Induced Cell Cycle Arrest and Apoptosis by Cyclin-Dependent Kinase Inhibitor p21/WAF1/CIP1. Cell Cycle.

[71] Roy S., Kaur M., Agarwal C., Tecklenburg M., Sclafani R.A., Agarwal R. (2007). p21 and p27 induction by silibinin is essential for its cell cycle arrest effect in prostate carcinoma cells. Mol. Cancer Ther..

[72] Huang H.-C., Way T.-D., Lin C.-L., Lin J.-K. (2008). EGCG Stabilizes p27kip1 in E2-Stimulated MCF-7 Cells through Down-Regulation of the Skp2 Protein. Endocrinology.

[73] Einbond L.S., Wu H.-A., Kashiwazaki R., He K., Roller M., Su T., Wang X., Goldsberry S. (2012). Carnosic acid inhibits the growth of ER-negative human breast cancer cells and synergizes with curcumin. Fitoterapia.

[74] Mahmoud N., Saeed M.E., Sugimoto Y., Klinger A., Fleischer E., Efferth T. (2020). Putative molecular determinants mediating sensitivity or resistance towards carnosic acid tumor cell responses. Phytomedicine.

[75] Zhang X., Chen Y., Cai G., Li X., Wang D. (2017). Carnosic acid induces apoptosis of hepatocellular carcinoma cells via ROS-mediated mitochondrial pathway. Chem. Biol. Interact..

[76] Su K., Wang C.-F., Zhang Y., Cai Y.-J., Zhang Y.-Y., Zhao Q. (2016). The inhibitory effects of carnosic acid on cervical cancer cells growth by promoting apoptosis via ROS-regulated signaling pathway. Biomed. Pharmacother..

[77] Kim D.-H., Park K.-W., Chae I.G., Kundu J., Kim E.-H., Kundu J.K., Chun K.-S. (2016). Carnosic acid inhibits STAT3 signaling and induces apoptosis through generation of ROS in human colon cancer HCT116 cells. Mol. Carcinog..

[78] Fantini M., Benvenuto M., Masuelli L., Frajese G.V., Tresoldi I., Modesti A., Bei R. (2015). In Vitro and in Vivo Antitumoral Effects of Combinations of Polyphenols, or Polyphenols and Anticancer Drugs: Perspectives on Cancer Treatment. Int. J. Mol. Sci..

[79] Chen X., Li H., Zhang B., Deng Z. (2021). The synergistic and antagonistic antioxidant interactions of dietary phytochemical combinations. Crit. Rev. Food Sci. Nutr..

[80] Nabekura T., Yamaki T., Hiroi T., Ueno K., Kitagawa S. (2010). Inhibition of anticancer drug efflux transporter P-glycoprotein by rosemary phytochemicals. Pharmacol. Res..

[81] Li H., Krstin S., Wink M. (2018). Modulation of multidrug resistant in cancer cells by EGCG, tannic acid and curcumin. Phytomedicine.

[82] Costea T., Vlad O.C., Miclea L.-C., Ganea C., Szöllősi J., Mocanu M.-M. (2020). Alleviation of Multidrug Resistance by Flavonoid and Non-Flavonoid Compounds in Breast, Lung, Colorectal and Prostate Cancer. Int. J. Mol. Sci..

[83] Nimiya Y., Wang W., Du Z., Sukamtoh E., Zhu J., Decker E., Zhang G. (2016). Redox modulation of curcumin stability: Redox active antioxidants increase chemical stability of curcumin. Mol. Nutr. Food Res..

[84] Hope-Roberts M., Horobin R.W. (2017). A review of curcumin as a biological stain and as a self-visualizing pharmaceutical agent. Biotech. Histochem..

[85] Levine C.B., Bayle J., Biourge V., Wakshlag J.J. (2017). Cellular effects of a turmeric root and rosemary leaf extract on canine neoplastic cell lines. BMC Veter. Res..

[86] Sapandowski A., Stope M.B., Evert K., Evert M., Zimmermann U., Peter D., Päge I., Burchardt M., Schild L. (2015). Cardiolipin composition correlates with prostate cancer cell proliferation. Mol. Cell. Biochem..

[87] Ben-Zichri S., Kolusheva S., Danilenko M., Ossikbayeva S., Stabbert W.J., Poggio J.L., Stein D.E., Orynbayeva Z., Jelinek R. (2019). Cardiolipin mediates curcumin interactions with mitochondrial membranes. Biochim. Biophys. Acta BBA Biomembr..

[88] Lim H.W., Lim H.Y., Wong K.P. (2009). Uncoupling of oxidative phosphorylation by curcumin: Implication of its cellular mechanism of action. Biochem. Biophys. Res. Commun..

[89] Nazıroğlu M., Çiğ B., Yazğan Y., Schwaerzer G.K., Theilig F., Pecze L. (2019). Albumin evokes Ca^2+^-induced cell oxidative stress and apoptosis through TRPM2 channel in renal collecting duct cells reduced by curcumin. Sci. Rep..

[90] Moustapha A., Peretout P.A., Rainey N.E., Sureau F., Geze M., Petit J.M., Dewailly E., Slomianny C., Petit P.X. (2015). Curcumin induces crosstalk between autophagy and apoptosis mediated by calcium release from the endoplasmic reticulum, lysosomal destabilization and mitochondrial events. Cell Death Discov..

[91] Keer H.N., Gaylis F.D., Kozlowski J.M., Kwaan H.C., Bauer K.D., Sinha A.A., Wilson M.J. (1991). Heterogeneity in plasminogen activator (PA) levels in human prostate cancer cell lines: Increased PA activity correlates with biologically aggressive behavior. Prostate.

[92] O’Keeffe B.A., Cilia S., Maiyar A.C., Vaysberg M., Firestone G.L. (2013). The serum- and glucocorticoid-induced protein kinase-1 (Sgk-1) mitochondria connection: Identification of the IF-1 inhibitor of the F1F0-ATPase as a mitochondria-specific binding target and the stress-induced mitochondrial localization of endogenous Sgk-1. Biochimie.

[93] Zhu R., Yang G., Cao Z., Shen K., Zheng L., Xiao J., You L., Zhang T. (2020). The prospect of serum and glucocorticoid-inducible kinase 1 (SGK1) in cancer therapy: A rising star. Ther. Adv. Med Oncol..

[94] Fagerli U.-M., Ullrich K., Stühmer T., Holien T., Köchert K., Holt R.U., Bruland Ø.S., Chatterjee M., Nogai H., Lenz G. (2011). Serum/glucocorticoid-regulated kinase 1 (SGK1) is a prominent target gene of the transcriptional response to cytokines in multiple myeloma and supports the growth of myeloma cells. Oncogene.

[95] Sahoo S., Brickley D.R., Kocherginsky M., Conzen S.D. (2005). Coordinate expression of the PI3-kinase downstream effectors serum and glucocorticoid-induced kinase (SGK-1) and Akt-1 in human breast cancer. Eur. J. Cancer.

[96] Liu W., Wang X., Wang Y., Dai Y., Xie Y., Ping Y., Yin B., Yu P., Liu Z., Duan X. (2018). SGK1 inhibition-induced autophagy impairs prostate cancer metastasis by reversing EMT. J. Exp. Clin. Cancer Res..

[97] Sherk A.B., Frigo D., Schnackenberg C.G., Bray J.D., Laping N.J., Trizna W., Hammond M., Patterson J.R., Thompson S.K., Kazmin D. (2008). Development of a Small-Molecule Serum- and Glucocorticoid-Regulated Kinase-1 Antagonist and Its Evaluation as a Prostate Cancer Therapeutic. Cancer Res..

[98] Liu W., Wang X., Liu Z., Wang Y., Yin B., Yu P., Duan X., Liao Z., Chen Y., Liu C. (2017). SGK1 inhibition induces autophagy-dependent apoptosis via the mTOR-Foxo3a pathway. Br. J. Cancer.

[99] Seo S.U., Woo S.M., Lee H.-S., Kim S.H., Min K.-J., Kwon T.K. (2018). mTORC1/2 inhibitor and curcumin induce apoptosis through lysosomal membrane permeabilization-mediated autophagy. Oncogene.

[100] Catalogna G., Moraca F., D’Antona L., Dattilo V., Perrotti G., Lupia A., Costa G., Ortuso F., Iuliano R., Trapasso F. (2019). Review about the multi-target profile of resveratrol and its implication in the SGK1 inhibition. Eur. J. Med. Chem..

[101] Qin J., Chen J.X., Zhu Z., Teng J.A. (2015). Genistein Inhibits Human Colorectal Cancer Growth and Suppresses MiR-95, Akt and SGK1. Cell. Physiol. Biochem..

[102] Liang X., Lan C., Jiao G., Fu W., Long X., An Y., Wang K., Zhou J., Chen T., Li Y. (2017). Therapeutic inhibition of SGK1 suppresses colorectal cancer. Exp. Mol. Med..

[103] Bray F., Ferlay J., Soerjomataram I., Siegel R.L., Torre L.A., Jemal A. (2018). Global cancer statistics 2018: GLOBOCAN estimates of incidence and mortality worldwide for 36 cancers in 185 countries. CA Cancer J. Clin..

[104] Rebello R.J., Oing C., Knudsen K.E., Loeb S., Johnson D.C., Reiter R.E., Gillessen S., Van der Kwast T., Bristow R.G. (2021). Prostate cancer. Nat. Rev. Dis. Primers.

