# Supplementary material for: Curcumin and Carnosic Acid Cooperate to Inhibit Proliferation and Alter Mitochondrial Function of Metastatic Prostate Cancer Cells
Source: Antioxidants (Basel). 2021 Oct 11;10(10):1591. doi: 10.3390/antiox10101591 (PMC8533243; doi:10.3390/antiox10101591)
Supplement: Supplementary file 1 [file antioxidants-10-01591-s001.zip › antioxidants-1295651-supplementary.pdf]

Supplementary Materials

Curcumin and Carnosic Acid Cooperate to Inhibit Proliferation and Alter Mitochondrial Function of Metastatic Prostate Cancer Cells

Saniya Ossikbayeva [1,2,†], Marina Khanin [3], Yoav Sharoni [3], Aviram Trachtenberg [3], Sultan Tuleukhanov [2], Richard Sensenig [4], Slava Rom [5], Michael Danilenko [3,*,‡] and Zulfiya Orynbayeva [1,‡,§]

[1] Department of Surgery, Drexel University College of Medicine, Philadelphia, PA 19102, USA; ossikbayeva.s@kaznmu.kz (S.O.); zulfiya.orynbayeva@crl.com (Z.O.)

[2] Department of Biophysics and Biomedicine, Al-Farabi Kazakh National University, Almaty 050040, Kazakhstan; sultan.tuleuhanov@kaznu.kz

[3] Department of Clinical Biochemistry and Pharmacology, Faculty of Health Sciences, Ben-Gurion University of the Negev, Beer Sheva 8410501, Israel; hanin@bgu.ac.il (M.K.); yoav@bgu.ac.il (Y.S.); aviramtr@post.bgu.ac.il (A.T.)

[4] Department of Surgery, Cooper University Hospital, Camden, NJ 08103, USA; sensenigrb@verizon.net

[5] Department of Pathology and Laboratory Medicine, Lewis Katz School of Medicine, Temple University, Philadelphia, PA 19140, USA; srom@temple.edu

* Correspondence: misha@bgu.ac.il; Tel.: +972-8-647-9969

† Current address: Kazakh Research Institute of Oncology and Radiology, Almaty 050022, Kazakhstan.

‡ Equal contribution.

§ Current address: Charles River Laboratories, Malvern, PA 19355, USA.

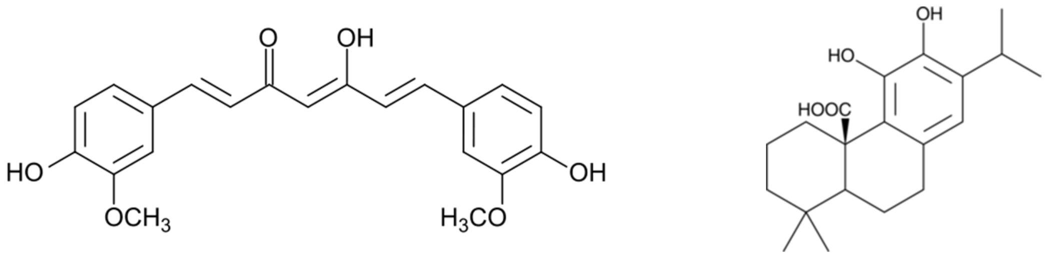

Curcumin **Carnosic acid**

Figure S1. Chemical structures of curcumin and carnosic acid

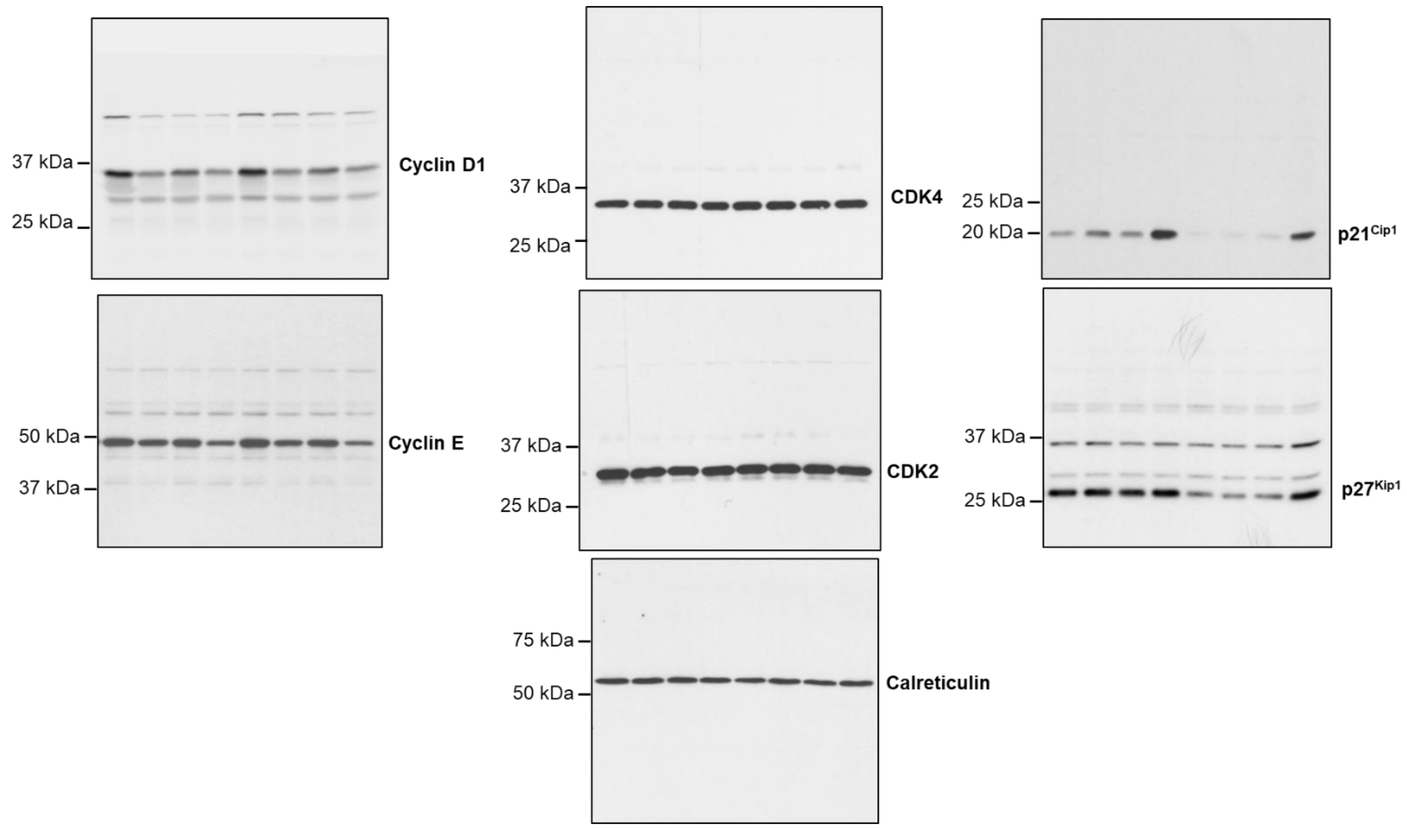

Figure S2. Original images of the Western blots shown in Figure 4c

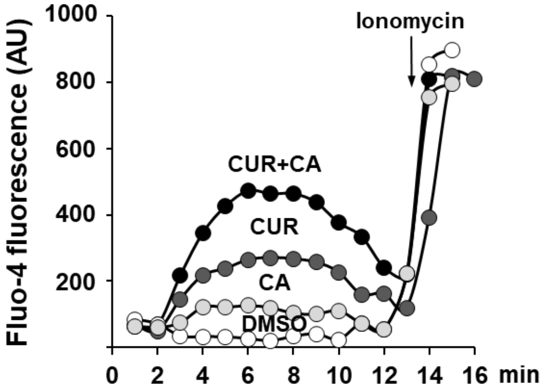

Figure S3. Curcumin and carnosic acid cooperate in inducing moderate and transient cytosolic calcium elevation in DU145 prostate cancer cells. Fluo-4 emission was recorded kinetically at 1-min intervals, starting immediately upon the addition of 7 μM curcumin (CUR), 5 μM carnosic acid (CA) their combination (CUR+CA) and the vehicle DMSO. Note that while CA alone has only a modest effect on $[Ca^{2+}]_{cyt}$, CUR and, particularly, its combination with CA produce an appreciable elevation of $[Ca^{2+}]_{cyt}$ that reaches 30-40% of the maximal response induced by 10 μM ionomycin.

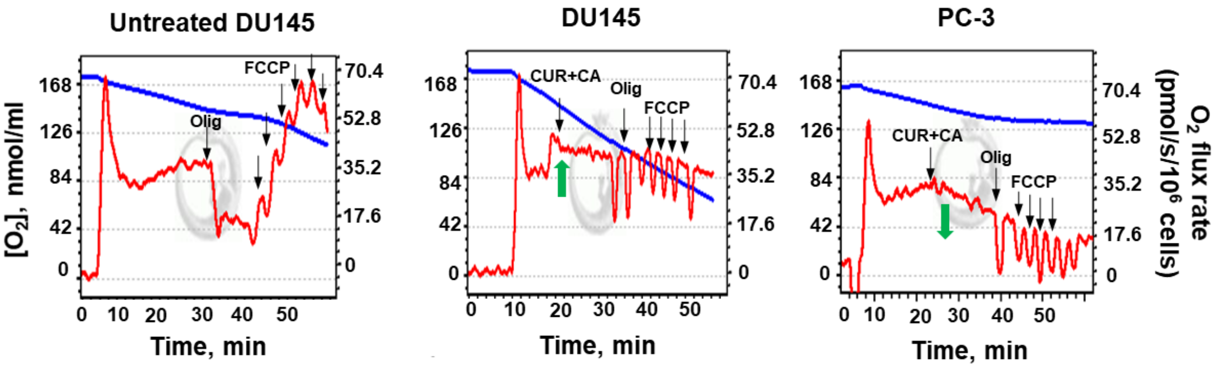

Figure S4. Combinatory effects of curcumin and carnosic acid on mitochondria respiration. Respirogram of the oxygen consumption by intact untreated DU145 cells exemplifying the respirometric protocol applied to all the cell types tested. Original respirograms for three types of intact cells are recorded in the presence of 7 μM CUR and 5 μM CA (CUR+CA). Abbreviations: *Olig*, 1 μg/ml oligomycin-inhibited residual respiration; *FCCP*, stimulation with 20 nM steps of FCCP to obtain the maximal respiratory capacity of mitochondria.

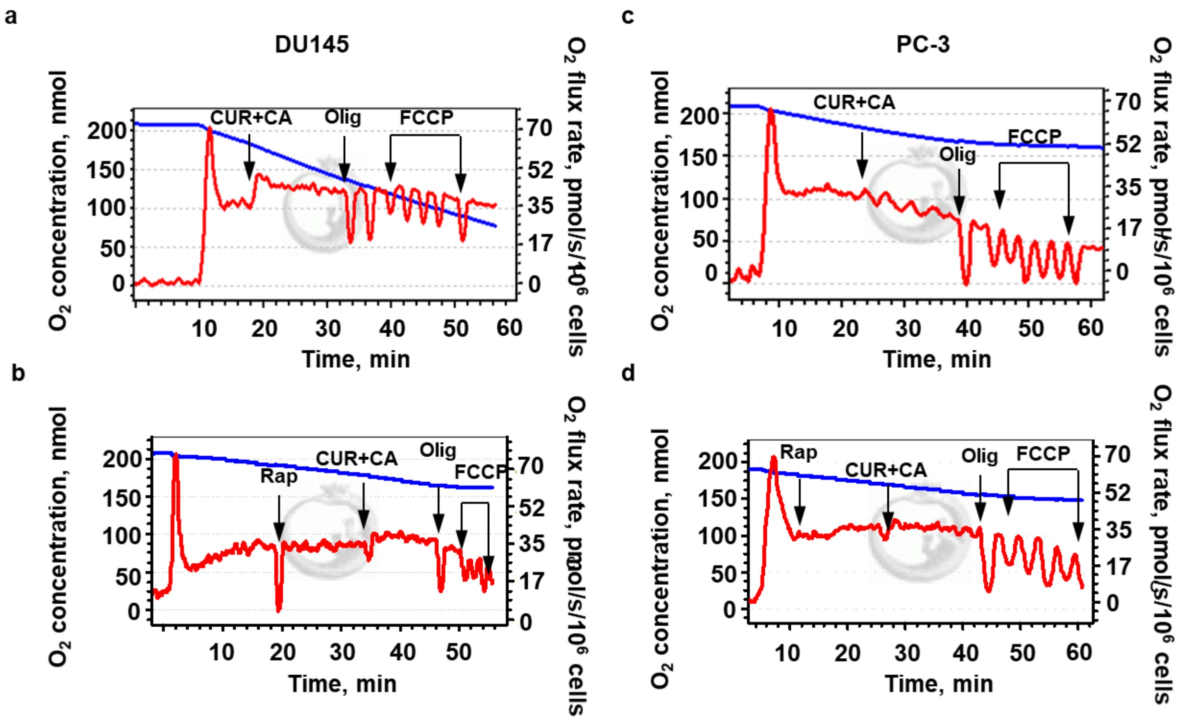

Figure S5. Oxygraphic records of the effects of the combination of curcumin and carnosic acid on respiration of prostate cancer cells, without pretreatment (**a, c**) or following pre-administration of 5 μM rapamycin (**b, d**). See the legend to Figure S4 for details.

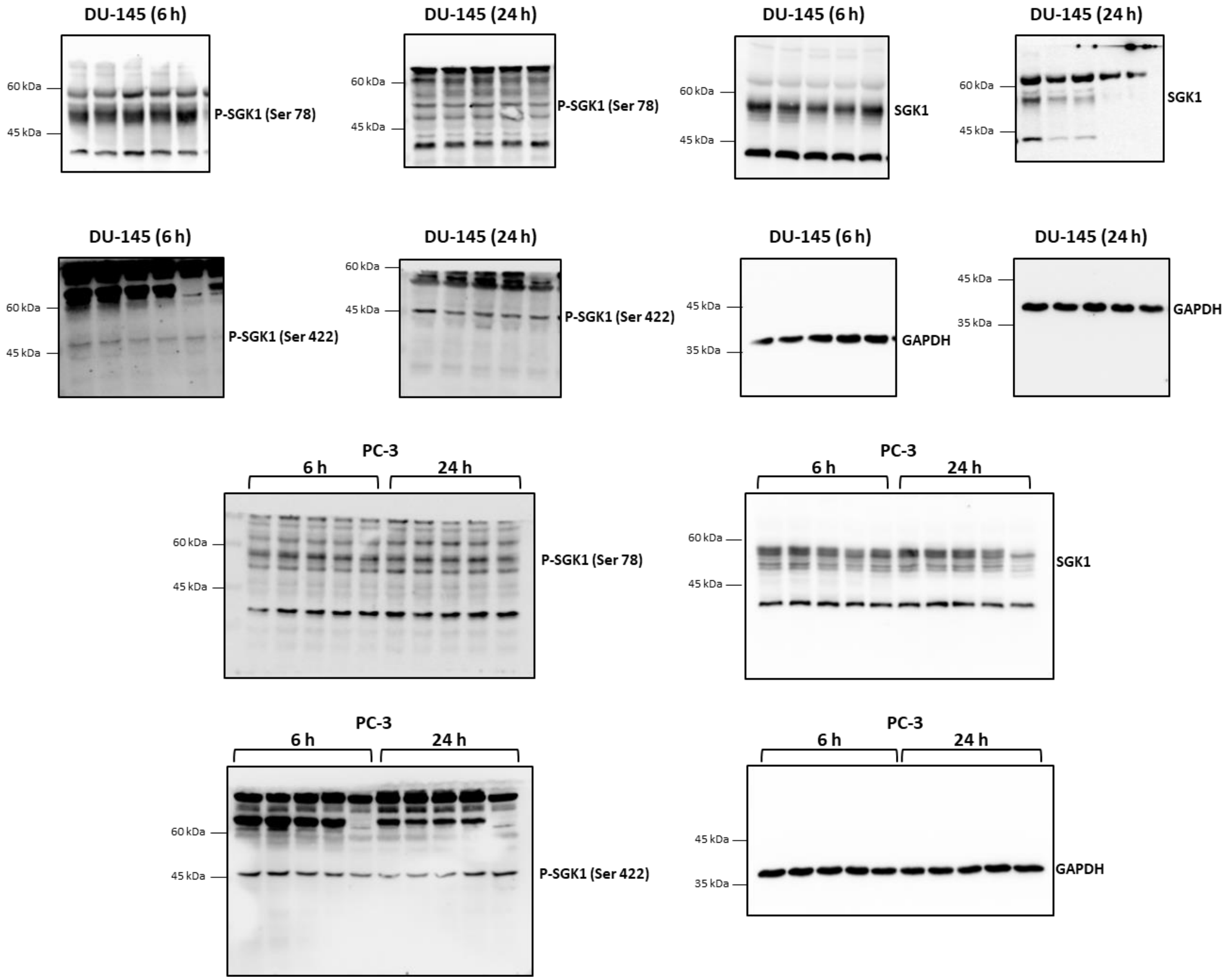

Figure S6. Original images of the Western blots shown in Figure 10
